# WJMSC‐derived small extracellular vesicle enhance T cell suppression through PD‐L1

**DOI:** 10.1002/jev2.12067

**Published:** 2021-02-08

**Authors:** Meizhang Li, Rupal Soder, Sunil Abhyankar, Haitham Abdelhakim, Mitchell W. Braun, Camille V. Trinidad, Harsh B. Pathak, Ziyan Pessetto, Clayton Deighan, Siddhartha Ganguly, Buddhadeb Dawn, Joseph McGuirk, Neil Dunavin, Andrew K. Godwin

**Affiliations:** ^1^ Department of Pathology and Laboratory Medicine University of Kansas Medical Center Kansas City Kansas USA; ^2^ Midwest Stem Cell Therapy Center University of Kansas Medical Center Kansas City Kansas USA; ^3^ Division of Hematologic Malignancies and Cellular Therapeutics University of Kansas Medical Center Kansas City Kansas USA; ^4^ The University of Kansas Cancer Center, University of Kansas Medical Center Kansas City Kansas USA; ^5^ Department of Microbiology Molecular Genetics and Immunology Kansas City Kansas USA; ^6^ NanoView Biosciences Boston Massachusetts USA; ^7^ Department of Medicine University of Nevada Las Vegas Nevada USA; ^8^ Division of Hematology and Blood and Marrow Transplant University of California San Francisco San Francisco California USA

**Keywords:** acute graft‐versus‐host disease, PD‐L1, small extracellular vesicles, T cell receptor, Wharton's Jelly‐derived mesenchymal stem cells

## Abstract

Both mesenchymal stem cells (MSCs) and their corresponding small extracellular vesicles (sEVs, commonly referred to as exosomes) share similar immunomodulatory properties that are potentially beneficial for the treatment of acute graft versus host disease (aGvHD). We report that clinical grade Wharton's Jelly‐derived MSCs (WJMSCs) secrete sEVs enriched in programmed death‐ligand 1 (PD‐L1), an essential ligand for an inhibitory immune checkpoint. A rapid increase in circulating sEV‐associated PD‐L1 was observed in patients with aGvHD and was directly associated with the infusion time of clinical grade WJMSCs. In addition, in vitro inhibitory antibody mediated blocking of sEV‐associated PD‐L1 restored T cell activation (TCA), suggesting a functional inhibitory role of sEVs‐PD‐L1. PD‐L1‐deficient sEVs isolated from WJMSCs following CRISPR‐Cas9 gene editing fail to inhibit TCA. Furthermore, we found that PD‐L1 is essential for WJMSC‐derived sEVs to modulate T cell receptors (TCRs). Our study reveals an important mechanism by which therapeutic WJMSCs modulate TCR‐mediated TCA through sEVs or sEV‐carried immune checkpoints. In addition, our clinical data suggest that sEV‐associated PD‐L1 may be not only useful in predicting the outcomes from WJMSC clinical administration, but also in developing cell‐independent therapy for aGvHD patients.

## 1 INTRODUCTION

Allogeneic hematopoietic cell transplantation (HCT) provides a potential cure for hematological malignancies, which depends in part on the immune graft‐versus‐leukemia (GvL) effects mediated by donor T cells (Chakraverty & Sykes, 2007; Ito & Shizuru, 1999). However, HCT may also cause the serious complication of immune acute graft‐versus‐host disease (aGvHD) due to donor T cells recognizing and attacking the recipient's non‐malignant tissues (Blazar et al., 2012; Ratanatharathorn et al., 2001; Zeiser & Blazar, 2017).

Programmed death ligand‐1 (PD‐L1) is a member of the B7 family, consisting of structurally related, cell‐surface proteins that regulate T cell activation (TCA) and tolerance through the T cell receptor (TCR) (Greaves & Gribben, 2013). Depending on its interaction with either receptor PD‐1 or CD80 expressed on the T cells, PD‐L1 can function differently as either a coinhibitory or costimulatory checkpoint signal for the T cells (Butte et al., 2007; Butte et al., 2008; Freeman et al., 2000; Fumihiko Tsushima et al., 2007; Keir et al., 2007). It has been suggested that donor CD8^+^ T cells expressing high levels of CD80 that leads in PD‐L1/CD80 interaction in lymphoid tissue with subsequent CD8^+^ T cell expansion to promote GvL effects (Cassady et al., 2018; Ni et al., 2017). However, in non‐malignant target tissues, the binding of PD‐L1 to PD‐1 induces the anergy, exhaustion or apoptosis to establish donor's T cell tolerance and prevent GvHD (Cassady et al., 2018; Fujiwara et al., 2014; Kitazawa et al., 2007; Saha et al., 2016).

Mesenchymal stem cells (MSCs) have been widely applied to treat aGvHD (Dotoli et al., 2017; Kebriaei et al., 2020; Kurtzberg et al., 2020; Maziarz et al., 2015; Muroi et al., 2016), and one of their important mechanisms is their immunomodulatory effects related to PD‐L1 (Ankrum et al., 2014; Pittenger et al., 2019; Shi et al., 2018). In GvHD patients, infused MSCs inhibit the cellular proliferation and activation of CD4^+^ and CD8^+^ T‐cells through PD‐L1‐PD1 signalling (Zhang et al., 2020). The release of PD‐L1 from MSCs is regulated by pro‐inflammatory cytokines (Davies et al., 2017; Francisco et al., 2009; Guan et al., 2018) and is a pivotal ‘licensing’ step to maintain MSCs’ immunomodulatory capabilities (Carvalho et al., 2019; Krampera, 2011). The role of secretory factors is crucial to explain the immunomodulatory function for MSCs from the bone marrow in vivo, given that MSCs migrate to the lungs and cannot reach systemic circulation in humans after infusion (Barbash et al., 2003; Eggenhofer et al., 2012).

Extracellular vesicles (EVs) represent a heterogeneous group of lipid bilayer membranous structures, which originate from the endosomal system (Van Niel et al., 2018). Small EVs (sEVs, also referred to as exosomes) usually have an average size between 30–150 nm. These sEVs can communicate with other cells and reshape surrounding microenvironments by transporting and delivering bioactive molecules (Barile & Vassalli, 2017). It has been demonstrated that tumour‐associated exosomes suppress anti‐tumour immune activities through inhibitory PD‐L1‐PD1 (Boussiotis, 2016; Daassi et al., 2020). For example, exosomal PD‐L1 inhibit CD8^+^ T cells activation associated with anti‐PD1‐PD‐L1 therapy (Chen et al., 2018; Poggio et al., 2019; Ricklefs et al., 2018), and, if exosomal PD‐L1 is suppressed, systemic anti‐tumour immunity and memory will be recovered (Poggio et al., 2019; Ricklefs et al., 2018). Recent studies also have found that MSC‐secreted exosomes inhibited the activation of CD4^+^ and CD8^+^ T cells (Fujii et al., 2018; Lai et al., 2018) and induced the differentiation of regulatory T cells (Ankrum et al., 2014; Dotoli et al., 2017; Maziarz et al., 2015; Muroi et al., 2016; Shi et al., 2018). Like tumoral exosomes, MSC‐associated exosomes have been suggested to be an attractive therapeutic agent for the treatment of immune‐mediated disorders through immunomodulation of T cell function (Gomzikova et al., 2019); however clinical trials have resulted in variable success and an optimal source of MSC has yet to be defined. Furthermore, there is currently no direct evidence for the intrinsic connection between MSC therapy and their associated exosomes, though MSC exosomes potentially contribute to the treatment of GvHDs (Fujii et al., 2018; Kordelas et al., 2014; Lai et al., 2018). For instance, a recent clinical study has shown that MSC‐derived exosomes can effectively improve GvHD symptoms by reducing the pro‐inflammatory cytokine response of donor PBMCs (Kordelas et al., 2014). However, their roles in regulating T cell function, specifically the profiling of checkpoint protein components on sEVs which underly the molecular mechanisms of sEV‐mediated therapy, have not been extensively explored.

In this study, we determined that PD‐L1 was enriched on the surface of WJMSC sEVs. The focus on WJMSC was based on the importance of maternal‐fetal interface immune tolerance, extraembryonic fetal tissues, such as the umbilical cord, and our recent clinical study which assessed whether this was a superior tissue source of MSC to mediate immunomodulation in aGVHD (Soder et al., 2020). To support this clinical trial, we investigated the role of WJMSC sEV‐PD‐L1 on TCR‐mediated TCA. We show that sEV‐PD‐L1 is necessary for the WJMSC‐mediated blockage of TCR‐mediated TCA. Mechanistically, membrane‐bound PD‐L1 provided by WJMSC‐associated sEVs is required for the regulation of pZAP70‐NFAT signalling pathway downstream of TCR. Finally, we show that increased circulation of sEV‐PDL1 in aGvHD patients is associated with WJMSC infusion. Our work provides insight into the molecular mechanisms through which MSC‐secreted PD‐L1^+^ sEVs target pathological immune cells related to aGvHD.

## 2 RESULTS

### 2.1 Identifying checkpoint PD‐L1 on WJMSC‐derived sEVs

**FIGURE 1:**
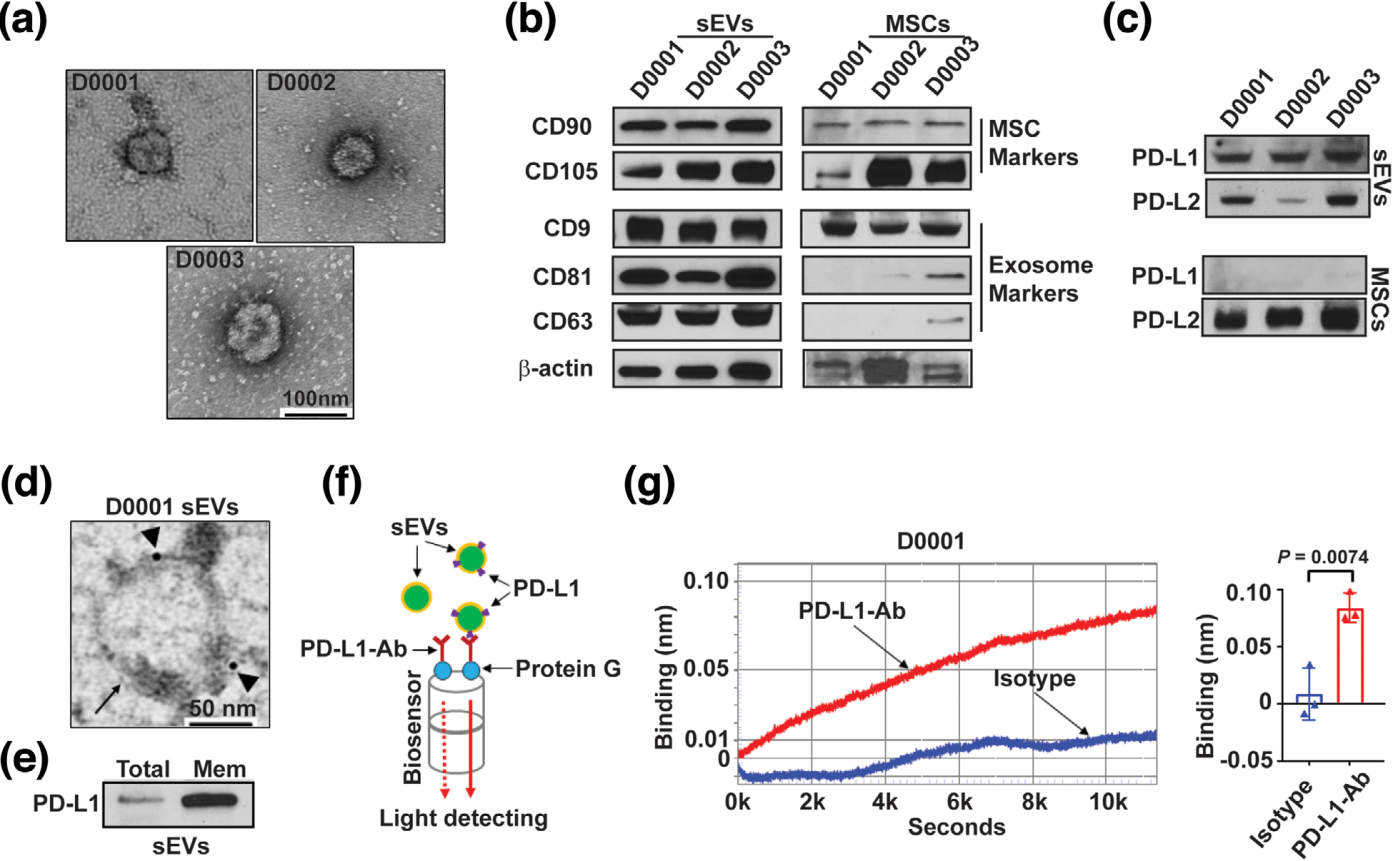
PD‐L1 is enriched on WJMSC‐derived sEVs. (a) Representative TEM imaging of purified sEVs from three clinical grade human WJMSCs (D0001, D0002 and D0003). Scale bar, 100 nm. (b‐c) Immunoblotting for stem cell biomarkers and exosome biomarkers (b), and checkpoint PD‐L1 and PD‐L2 (c) in purified WJMSC sEVs and the whole cell lysates (MSCs). All lanes were loaded with the same amount of protein. (d) A representative TEM image showing the immunogold‐labelled PD‐L1 signals (arrowheads) on the surface of a WJMSC sEV (arrow). Scale bar, 50 nm. (e) Immunoblotting for PD‐L1 in the whole exosome lysates (Total) and exosomal membrane protein extracts (Mem). All lanes were loaded with the same amount of protein. (f) Schematic of optical biolayer interferometry (BLI) assay for detecting surface PD‐L1 on the WJMSC sEVs. PD‐L1‐Ab, PD‐L1 detection antibody. (g) A typical binding (left) between PD‐L1‐Ab and WJMSC sEVs (red curve) or between Isotype and WJMSC sEVs (blue curve) and quantification analysis based on their wavelength differences (right, *n* = 3). Data are mean ± s.e.m. and analysed by unpaired one‐tailed Student's t‐test

Clinical grade WJMSCs (MSCTC‐0010) were manufactured by the Midwest Stem Cell Therapy Center (MSCTC) at the University of Kansas Medical Center (KUMC). These cells were used in a phase I study (NCT03158896) designed to evaluate the safety of MSCTC‐0010 in the treatment of de novo high risk acute or steroid refractory aGvHD (Soder et al., 2020). We demonstrated that WJMSCs express characteristic biomarkers such as CD90, CD105, and CD73 (Figure S1A) and maintain their multipotential under in vitro culture (Figure S1B). sEVs (30–150 nm size) were isolated from clinical grade WJMSCs using differential centrifugation (Figure S2A). Isolated WJMSC sEVs were characterized through Transmission Electron Microscopy (TEM, Figure 1a & Figure S2B) and Nanoparticle Tracking Analysis (NTA, Figure S2C). As expected, when compared with the cell lysate counterparts, WJMSC sEVs were enriched in conventional exosome‐associated biomarkers (e.g., CD9, CD63, and CD81) and the stem cell biomarker CD90 as analysed by immunoblotting (Figure 1b).

PD‐L1 was detected on the WJMSCs by flow cytometry (Figure S1C‐D) and surface PD‐L1 expression was confirmed on the WJMSC sEVs using immunogold TEM (Figure 1d). PD‐L1 was consistently enriched in the membrane protein extract isolated from WJMSC‐derived sEVs (Figure 1e). To confirm these findings, we used biolayer interferometry (BLI) assay to further detect and quantify surface PD‐L1 on sEVs using an anti‐human PD‐L1 antibody (Figure 1f). A measurable binding was observed between WJMSC sEVs and the PD‐L1 antibody (Figure 1g and Figure S3A‐C), supporting that checkpoint PD‐L1 was predominantly presented on the surface of WJMSC‐derived sEVs. To determine whether PD‐L1 was enriched on small EVs, we further applied qEVoriginal/70 nm columns (Izon Science, USA) to separate the exosomes from other EVs (Figure S3D‐E). We found that PD‐L1 was enriched on WJMSC‐derived exosomes using our custom PD‐L1/BLI assay (Figure S3F‐G).

### 2.2 WJMSC sEVs inhibit TCR‐mediated TCA through PD‐L1

MSC‐derived exosomes have been suggested to regulate the activation and differentiation of T cells (Chen et al., 2016; Zhang et al., 2018; Zhang et al., 2018). We investigated how WJMSC sEVs might modulate the activation of T cells mediated by T cell receptor (TCR). CD3/CD28 Dynabeads were used to stimulate peripheral blood mononuclear cells (PBMCs) in vitro, and subsequently, TCR‐mediated activation of CD4^+^ T cells was indicated by de novo CD154 up‐regulation (Figure S4A‐C). Increased protein expression of PD1 was found on activated CD4^+^ T cell surface (Figure S4D, F‐G) and directly correlated with CD154 expression (Figure S4E). There was 40 ± 2.5 % CD4^+^ T cells co‐expressed CD154 and PD1 (*n* = 6) after stimulation with CD3/CD28 Dynabeads (Figure S4F).

**FIGURE 2:**
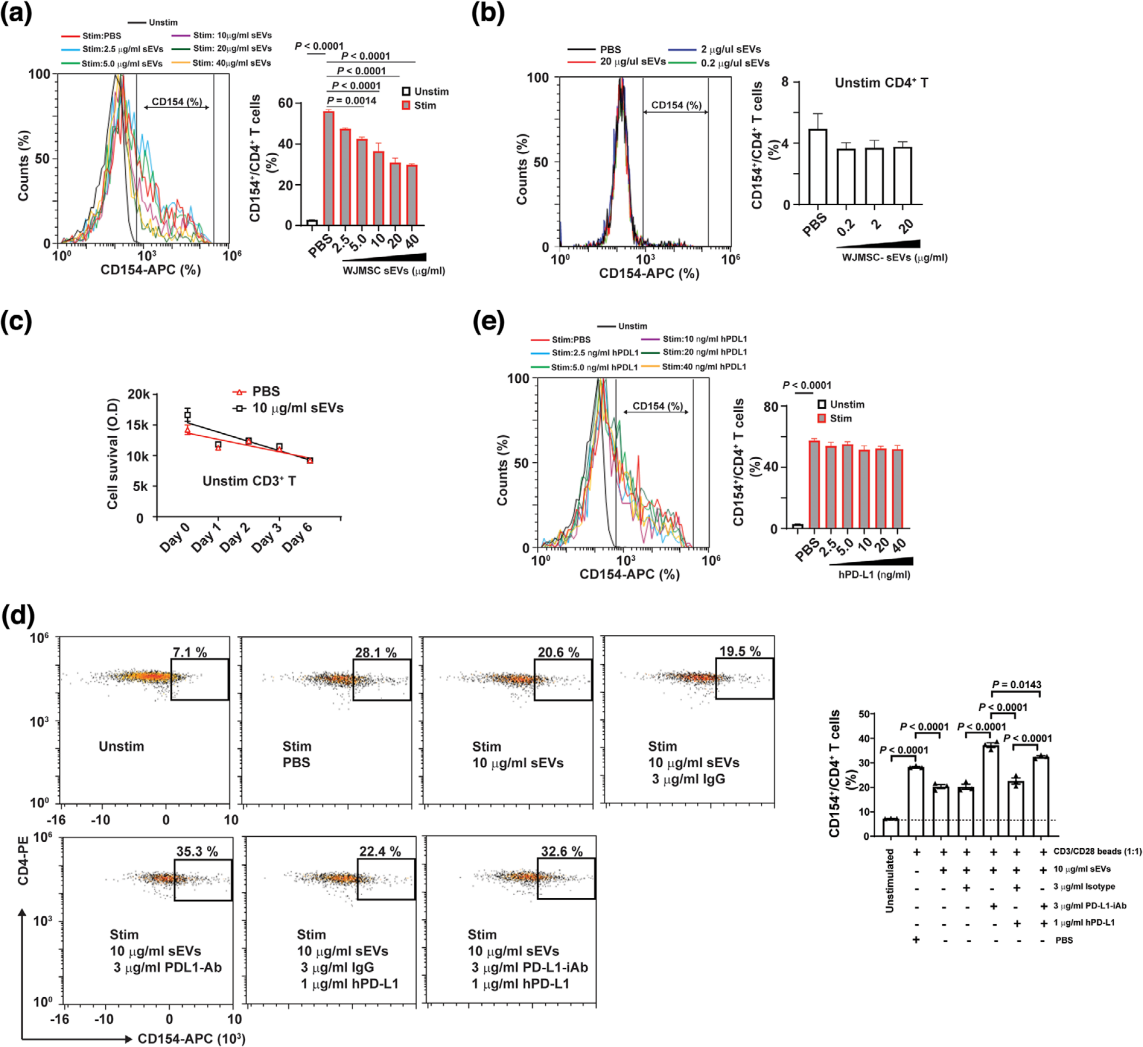
WJMSC sEVs inhibit TCR‐mediated CD4^+^ TCA through PD‐L1. (a) Flow cytometry showing the inhibitory effects of WJMSC‐derived sEVs on CD4^+^ T cell activation (CD154^+^) in a dose‐dependent manner (left) and quantitative analysis (right). (b) Effects of WJMSC sEVs on unstimulated CD4^+^ T cells (left) and quantitative analysis (right), measured by flow cytometry. (c) Survival of unstimulated CD3^+^ T cells treated with PBS or 10 µg/ml WJMSC sEVs for 6 days. (d) Representative flow charts (left) and quantitative analysis (right) of activated CD4^+^ T cells incubated with 10 µg/ml WJMSC sEVs, 3 µg/ml PD‐L1‐iAb/isotype, or 1 µg/ml hPD‐L1 for overnight. (e) Flow cytometry showing the effects of soluble hPD‐L1 on activated CD4^+^ T cells (left) and quantitative analysis (right). Peripheral blood mononuclear cell (PBMC)s were stimulated with (Unstim) or without (Stim) CD3/CD28 Dynabead at a dilution of 1:1 ratio (a,d,e). hPD‐L1, recombinant human PD‐L1 (d,e). PD‐L1‐iAb, neutralization antibody for human PD‐L1 (d). Data are mean ± s.e.m (*n* = 3) and analysed by one‐way ANOVA (a,b,d,e). Experiments independently repeated three times (a,d,e)

Next, we asked whether WJMSC‐derived sEVs affected the activation of CD4^+^ T cells stimulated by CD3/CD28 Dynabeads. We found that WJMSC sEVs significantly inhibited TCR‐mediated CD4^+^ T cell activation (TCA) in a dose‐dependent manner (Figure 2a). Normalized with the PBS, we found less expression of CD154 on CD4^+^ T cells 15 ± 1.2∼47 ± 0.8% (*n* = 3) after treating with 1.25∼20 µg/ml WJMSC sEVs for overnight. However, WJMSC sEVs were not able to either activate naïve CD4^+^ T cells (Figure 2b) or enhance their survival (Figure 2c). To confirm this observation, we investigated the effects of WJMSC‐derived sEVs on TCR‐mediated CD8^+^ TCA (Figure S5A‐B). Consistently, we found that WJMSC sEVs significantly (*P *< 0.05) inhibited the activation of CD8^+^ T cells (IFN‐γ^+^) stimulated by PepTivator cytomegalovirus (CMV) pp65 peptide (Figure S5C).

Exosomal PD‐L1 is critical for tumour cells to evade immune attack through coinhibitory regulation mechanisms related to the TCRs (Chen et al., 2018; Ricklefs et al., 2018). Because PD‐L1 was also enriched on the WJMSC‐derived sEVs, we asked whether WJMSC sEV‐PD‐L1 molecularly regulates TCR‐mediated CD4^+^ TCA. After treatment with 10 µg/ml sEVs overnight, TCR‐mediated TCA was reduced by 28 ± 6% (*n* = 3, *P* < 0.0001), and pre‐incubating sEVs with 3 mg/ml neutralization antibody can eliminate their inhibitory effects (Figure 2d). This loss of inhibition can be partially rescued by the addition of soluble human recombinant hPD‐L1 protein. However, we did not observe the direct effect of recombinant PD‐L1 on the activated CD4^+^ T cells (Figure 2e). Our results support that WJMSC‐derived sEVs inhibit TCA by modulating TCR and that PD‐L1 is involved in sEV‐mediated immunosuppression.

### 2.3 Genetic disruption of PD‐L1 impairs WJMSC sEVs’ capability to inhibit TCR‐mediated TCA

**FIGURE 3:**
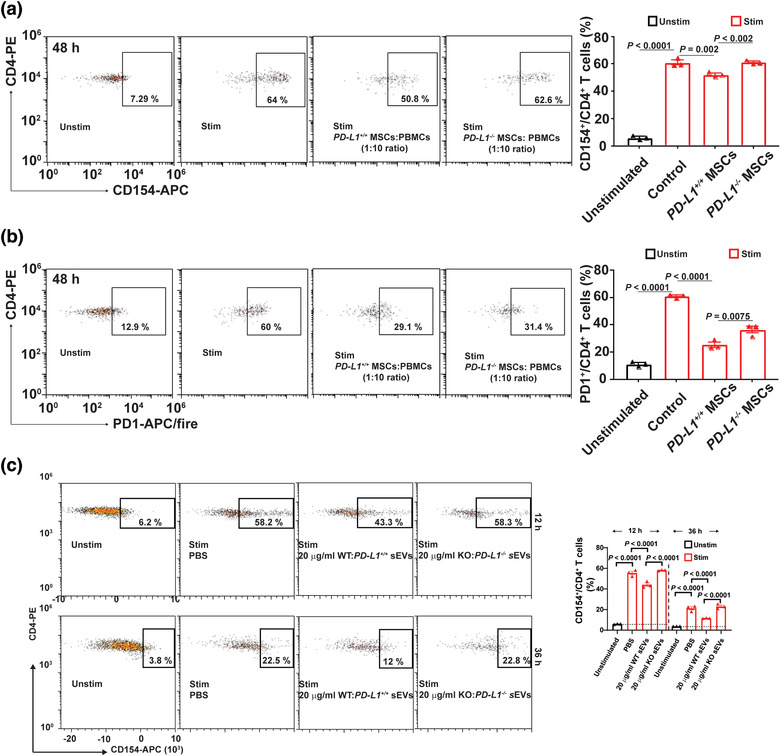
Both WJMSCs and sEVs decrease their capability to block TCR‐mediated TCA after PD‐L1 knockout. (a) Flow cytometry showing the 48‐hour inhibitory effects of both WT and KO WJMSCs on CD4^+^ T cell activation (left) and quantitative analysis (right). (b) Expression of PD1 on the CD4^+^ T cells mentioned above, measured by flow cytometry (left) and quantitation (right). (c) Representative flow charts (left) and quantitative analysis (right) of activated CD4^+^ T cells incubated with 20 µg/ml PD‐L1 WT or KO WJMSC sEVs for 12 h or 36 h. PBMCs were stimulated with (Unstim) or without (Stim) CD3/CD28 Dynabead at a dilution of 1:1 ratio (a‐c) and the ratio of WJMSCs to PBMCs was 1 to 10 (a,b). Data are mean ± s.e.m (*n* = 3) and analysed by one‐way ANOVA (a‐c). Data are representative of three independent experiments (a‐c)

To confirm the above observation, we generated PD‐L1 knockout (KO) WJMSCs by genetically deleting “‐CAGC‐” in the 3^rd^‐Exon through gene editing (Figure S6A). Loss of PD‐L1 protein in the *PD‐L1^–/–^* WJMSCs was verified by immunoblotting (Figure S6B) and flow cytometry (Figure S6C). These cells demonstrated the same normal phenotype as their wild types (Figure S6C‐D) and were not capable of expressing PD‐L1 after IFN‐γ treatment (Figure S6D‐E and Figure S7). The immune inhibitory effects of *PD‐L1^–/–^* WJMSCs on CD4^+^ TCA were further tested. We found *PD‐L1^–/–^* WJMSCs failed to block the CD4^+^ TCA compared with the WT WJMSCs by means of CD154 expression by flow cytometry (Figure 3a). Consistently, we found that expression of PD1 on activated CD4^+^ T cells was decreased by 58 ± 3% (*n* = 3, *P* < 0.0001) after treated with WT WJMSCs. Compared with the WT WJMSCs, expression of PD1 on activated CD4^+^ T cells was decreased by 28.6 ± 11% (*n* = 3, *P* < 0.0001) after treated with *PD‐L1^–/–^* WJMSCs (Figure 3b).

Next, sEVs were purified from *PD‐L1^–/–^* WJMSCs, and the loss of PD‐L1 on their corresponding sEVs was verified by immunoblotting (Figure S8A), TEM (Figure S8B), and BLI (Figure S8C). PD‐L1‐deficient sEVs demonstrate similar levels of the exo‐proteins, for example, CD81, PD‐L2, HSP70 (Figure S8A & F), morphology (Figure S8B & E), particle yield (Figure S8D) and average sizes (Figure S8D‐E) as their wild type counterparts. These sEVs were not induced by IFN‐γ to release PD‐L1 (Figure S8F‐G). Like WJMSCs, we show that PD‐L1‐deficient WJMSC sEVs significantly lost their capability to inhibit CD3/CD28 Dynabead‐stimulated CD4^+^ TCA (Figure 3c). Our results suggest that PD‐L1 carried by WJMSC sEVs are required for WJMSCs to inhibit TCR‐mediated TCA.

### 2.4 PD‐L1 carried by WJMSC sEVs is essential for modulating TCR's functions

**FIGURE 4:**
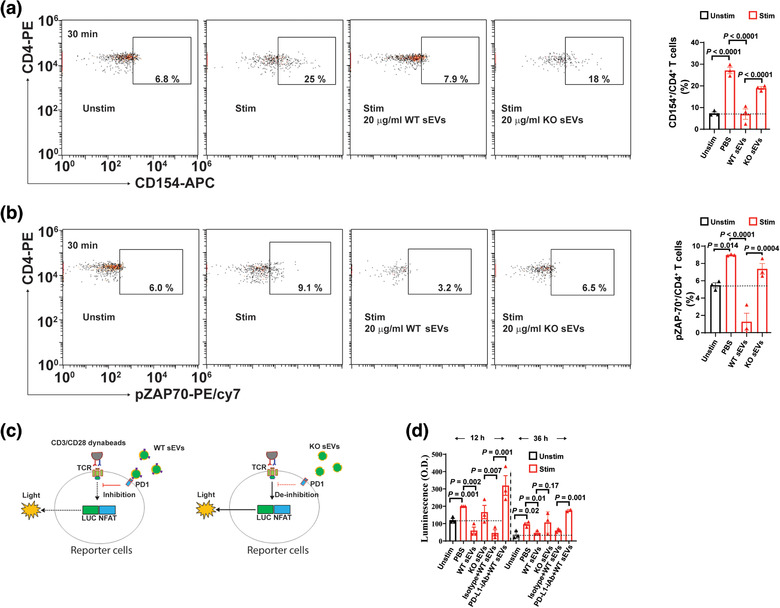
PD‐L1 is required for WJMSC sEVs to modulate TCR signalling pathway. (a) Representative flow charts showing the activation of CD4^+^ T cells incubated with 20 µg/ml PD‐L1 WT or KO WJMSC sEVs for 20 min (left) and quantitative analysis (right). (b) Expression of phospho‐ZAP70 protein (pZAP70) in activated CD4^+^ T cells mentioned above, measured by flow cytometry (left) and quantitation (right). (c) Schematic of PD1‐NFAT reporting system in Jurkat T cells stimulated with 1:1 ratio CD3/CD28 Dynabeads and co‐incubated with 20 µg/ml PD‐L1 WT and KO WJMSC sEVs plus 3 µg/ml PD‐L1‐iAb/isotype for 12 h or 36 h. LUCNFAT, luciferase gene under the control of NFAT response elements; PD‐L1‐iAb, neutralization antibody for human PD‐L1. (d) Quantitative analysis from c (*n* = 3). Data are mean ± s.e.m and analysed by one‐way ANOVA (a,b,d)

To determine whether WJMSC sEVs modulate the TCR signaling pathway through PD‐L1, we examined the protein level of phosphorylated zeta‐chain‐associated protein kinase 70 (pZAP70), a partner protein that is associated with activated TCRs, in T cells (Boussiotis, 2016; Gaud et al., 2018). In the activated CD4^+^ T cells (Figure 4a), we found that expression of pZAP70 was increased by 60 ± 10% (*n* = 3, *P* = 0.014) compared with the unstimulated CD4^+^ T cells (Figure 4b). Treating them with wild type WJMSC sEVs significantly decreased the pZAP70's expression by 89 ± 11% (*n* = 3, *P* < 0.0001) in the activated CD4^+^ T cells. However, treating them with PD‐L1‐deficient WJMSC sEVs only decreased the pZAP70's expression by 16 ± 5% (*n* = 3, *P* = 0.054) in the activated CD4^+^ T cells. Our results suggest that PD‐L1 carried by sEVs is critical for WJMSCs to inhibit CD4^+^ TCA through TCR.

Phosphorylation of ZAP70 protein induced its downstream nuclear translocation of nuclear factor of activated T cells (NFAT), signalling pathways through Ca^2+^–calcineurin (Boussiotis, 2016; Gaud et al., 2018). To determine whether WJMSC sEVs modulate this signalling pathway downstream of TCR, we examined their effect on Jurkat T cells that were engineered with a PD1‐NFAT‐luciferase reporter (Figure 4c). PD1‐NFAT reporter‐Jurkat cells were stimulated by CD3/CD28 Dynabeads (1:1 ratio) for 12 h and NFAT luciferase reporter activity was significantly increased by 69 ± 13% (*n* = 3, *P* = 0.001) compared with unstimulated reporter Jurkat cells (Figure 4d). Our results suggest that reporter‐Jurkat cells can be directly activated by CD3/CD28 Dynabeads and activation of NFAT signalling pathways is directly related to the stimulation of TCR. Next, we found that treating them with 20 µg/ml WT WJMSC sEVs significantly decreased the NFAT luciferase reporter activity by 70 ± 10% (*n* = 3, *P* = 0.002) in activated reporter‐Jurkat cells which could be reversed by neutralizing anti‐PD‐L1 antibody (Figure 4d). *PD‐L1^–/–^* WJMSCs derived sEVs did not cause inhibition of the activation of reporter‐Jurkat cells (Figure 4d). Similar results were obtained through stimulating and treating reporter‐Jurkat cells for 36 h. Our results support the concept that WJMSC sEVs modulate TCR function and regulate its downstream signalling pathway mainly through PD‐L1.

### 2.5 Increased plasma sEVs is associated with WJMSC infusion in aGvHD patients

**FIGURE 5:**
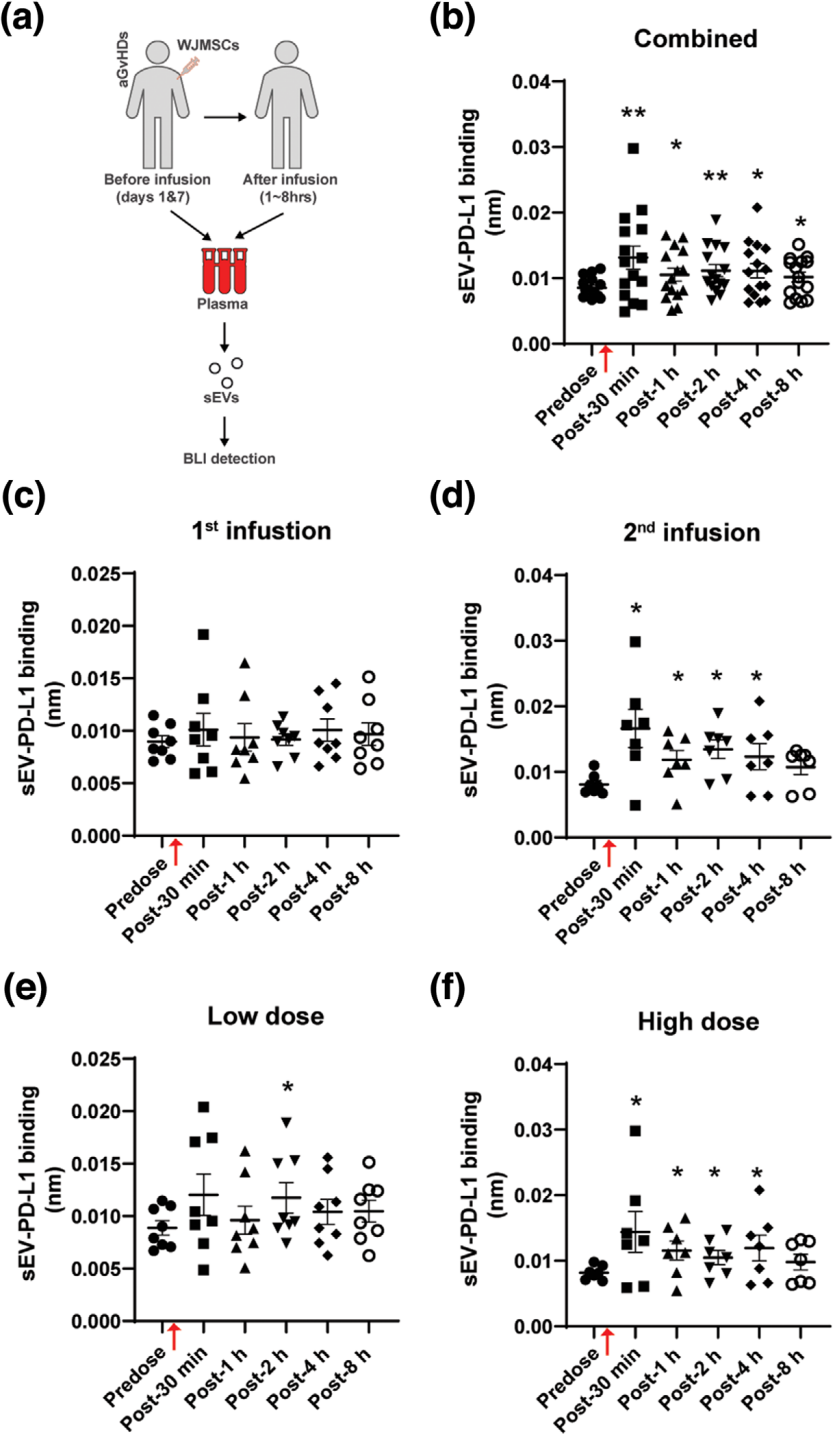
Clinical WJMSC infusion increases plasma sEV‐PD‐L1 in aGvHD patients. (a) Schematic of measuring plasma sEV‐PD‐L1 in aGvHD patients clinically received WJMSCs. Patients’ plasma samples were collected before (predose) and after (post) WJMSC transplantation within 8 h, sEVs were isolated from these plasma samples, and PD‐L1 on sEVs was examined by BLI. (b‐f) Quantitative analysis for combined patients (b, *n* = 15) and partial patients received with either 1^st^ infusion (c, *n* = 8), 2^nd^ infusion (d, *n* = 7), low dose: 1.2 × 10^6^ cells/kg (e, *n* = 8), or high dose: 10 × 10^6^ cells/kg (f, *n* = 7). WJMSC infusion time points were indicated by red arrows (b‐f). Data are mean ± s.e.m. and analysed by unpaired one‐tailed Student's t‐test (b‐f). ^*^ *P *< 0.05, ^**^ *P *< 0.01, ^***^ *P *< 0.005

To address whether WJMSCs might provide patients therapeutic benefit via sEV‐associated PD‐L1, we first applied BLI to examine the levels of sEV‐PD‐L1 in plasma samples from aGvHD patients infused with WJMSCs in a clinical trial at our center (Figure 5a). Compared to the baseline (pre‐dose) plasma level, the level of plasma sEV‐PD‐L1 was significantly increased (by 50%, *P *< 0.01) 30 min after WJMSC infusion (Figure 5b). A time‐dependent decline of plasma sEV‐PD‐L1 levels was observed at additional longitudinal screening points (from 1 to 8 h post infusion). Nevertheless, the circulating sEV‐PD‐L1 levels remained elevated when compared to the baseline level. In addition, we observed that multiple infusions (Figure 5c,d) and higher cell dosage (Figure 5e,f) more efficiently increased and maintained plasma levels of sEV‐PD‐L1 in aGvHD patients.

Next, we used ExoView™ chips (NanoView Bioscience, USA) to enumerate the quantity and population subtypes of sEVs in the plasma from these patients (Figure S9A). We found that total and CD81^+^ circulating sEVs captured by the CD81 antibody appeared significantly increased 1 h after WJMSC administration (Figure S9B‐C). Similar results were obtained from CD63‐ or CD9‐capture spots (Figure S9B & D‐E). All sEV subpopulations, including CD63^+^CD81^+^, CD63^+^CD9^+^, and CD9^+^CD81^+^, were observed to be significantly increased post‐infusion (Figure S9C‐F). Therefore, our results show that both increased circulation of sEVs and up‐regulation of sEV‐PD‐L1 are associated with WJMSC infusion in aGvHD patients. These findings suggest that WJMSC‐mediated immunomodulatory effects are related to increasing sEV‐PD‐L1 levels. Extending on recent studies indicating an immunomodulatory role of MSC‐derived exosomes in preventing GvHD (Kordelas et al., 2014; Lai et al., 2018), our results suggest for the first time that sEV‐PD‐L1 may represent a mediator of WJMSC immunosuppressive properties.

### 2.6 WJMSCs infusion demonstrated clinical responses in patients with advanced aGvHD

**FIGURE 6:**
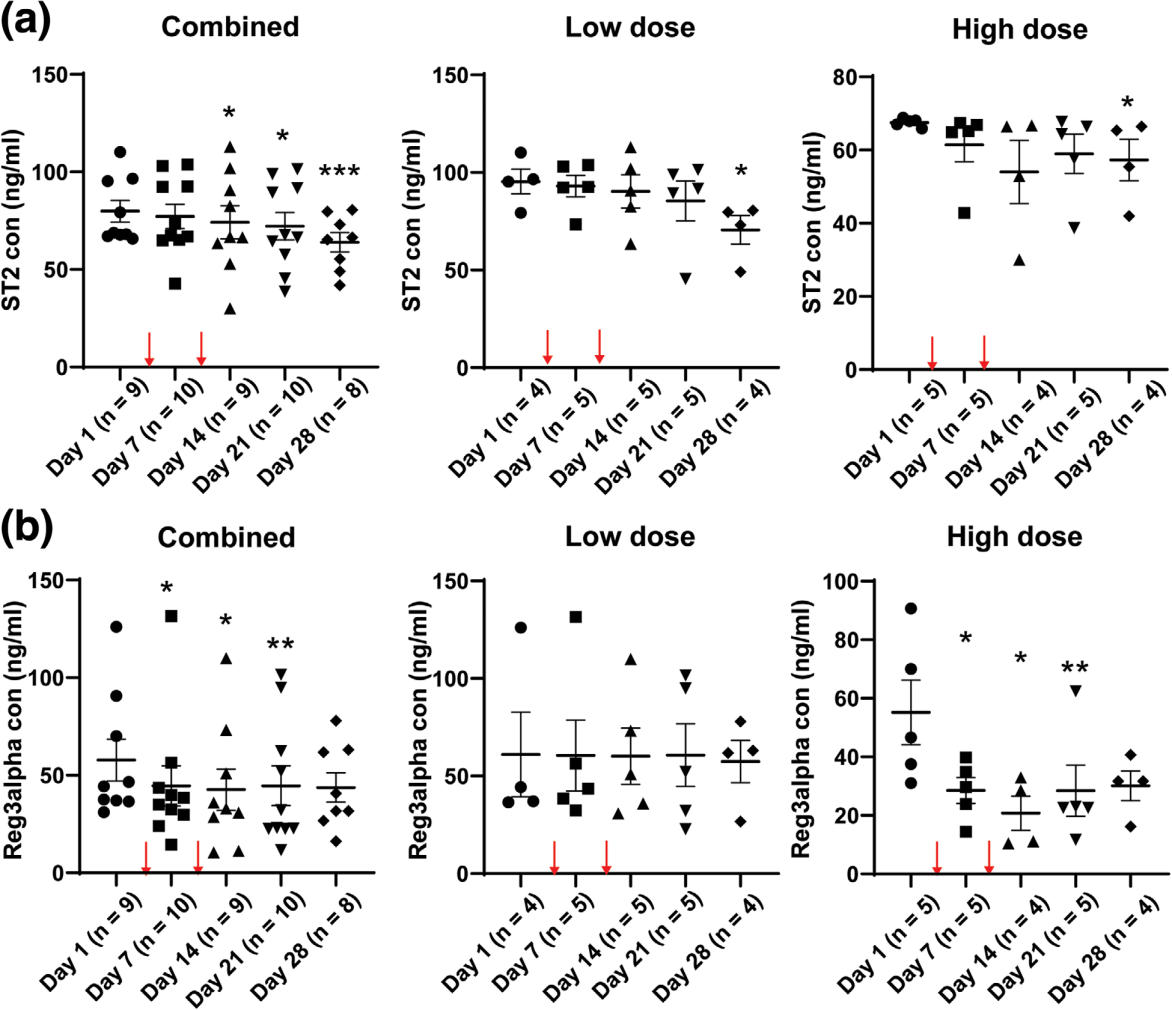
Decreased plasma ST2 and REG3A levels in aGvHD patients with WJMSC infusion. (a,b) ELISA of ST2 (a) and REG3A (b) in the plasma samples from aGvHD patients with the WJMSC infusion. Left, combined analysis; Middle, low‐dose (1.2 × 10^6^/kg WJMSCs); Right, high‐dose (10 × 10^6^/kg WJMSCs). Two cell infusion time points were indicated by red arrows (a,b). Data are mean ± s.e.m. and analysed by unpaired one‐tailed Student's t‐test (a,b). ^*^ *P *< 0.05, ^**^ *P *< 0.01, ^***^ *P *< 0.005

In the clinical trial (NCT03158896), 70% of patients with advanced aGvHD had an overall response including 40% of patients with a complete response thought to be attributable to MSCTC‐0010 infusions. This therapy was associated with median overall > 1 year in this high‐risk cohort (Soder et al., 2020). Patients on this trial did not show significant toxicities associated with treatment (Soder et al., 2020). To evaluate the potential therapeutic effects of WJMSCs in patients with aGvHD, we examined the plasma levels of two prognostic aGvHD biomarkers, that is, suppression of tumorigenicity 2 (ST2) and regenerating islet‐derived 3 alpha (REG3 alpha or REG3A) in plasma samples from patients with aGvHD (Dunavin et al., 2019; Levine et al., 2012; Lugt et al., 2013; Ponce et al., 2015; Rowan et al., 2020; Te Boome et al., 2015). We observed decreased plasma levels for both ST2 and REG3A (Figure 6a). Prior to WJMSC infusion (Day 1), the mean plasma ST2 level in aGvHD was 80 ng/ml. One week after the 2^nd^ WJMSC infusion (Day 14 from first infusion), we found that the mean plasma ST2 level in aGvHD patients decreased to 74 ng/ml, *P* < 0.05 (Figure A6, left). At post infusion day 21 and day 28, the mean plasma ST2 level decreased to 72 ng/ml (*P* < 0.05) and 64 ng/ml (*P* < 0.005), respectively. Consistently, we found that mean plasma REG3A levels decreased at days 7 (44.5 ng/ml, *P* < 0.05), 14 (42.7 ng/ml, *P* < 0.05) and 21 (44.6 ng/ml, *P* < 0.01) post WJMSCs infusion in aGvHD patients compared to pre‐infusion (57.8 ng/ml) (Figure 6b).

### 2.7 IFN‐γ induced the release of sEV‐PD‐L1 from WJMCs

**FIGURE 7:**
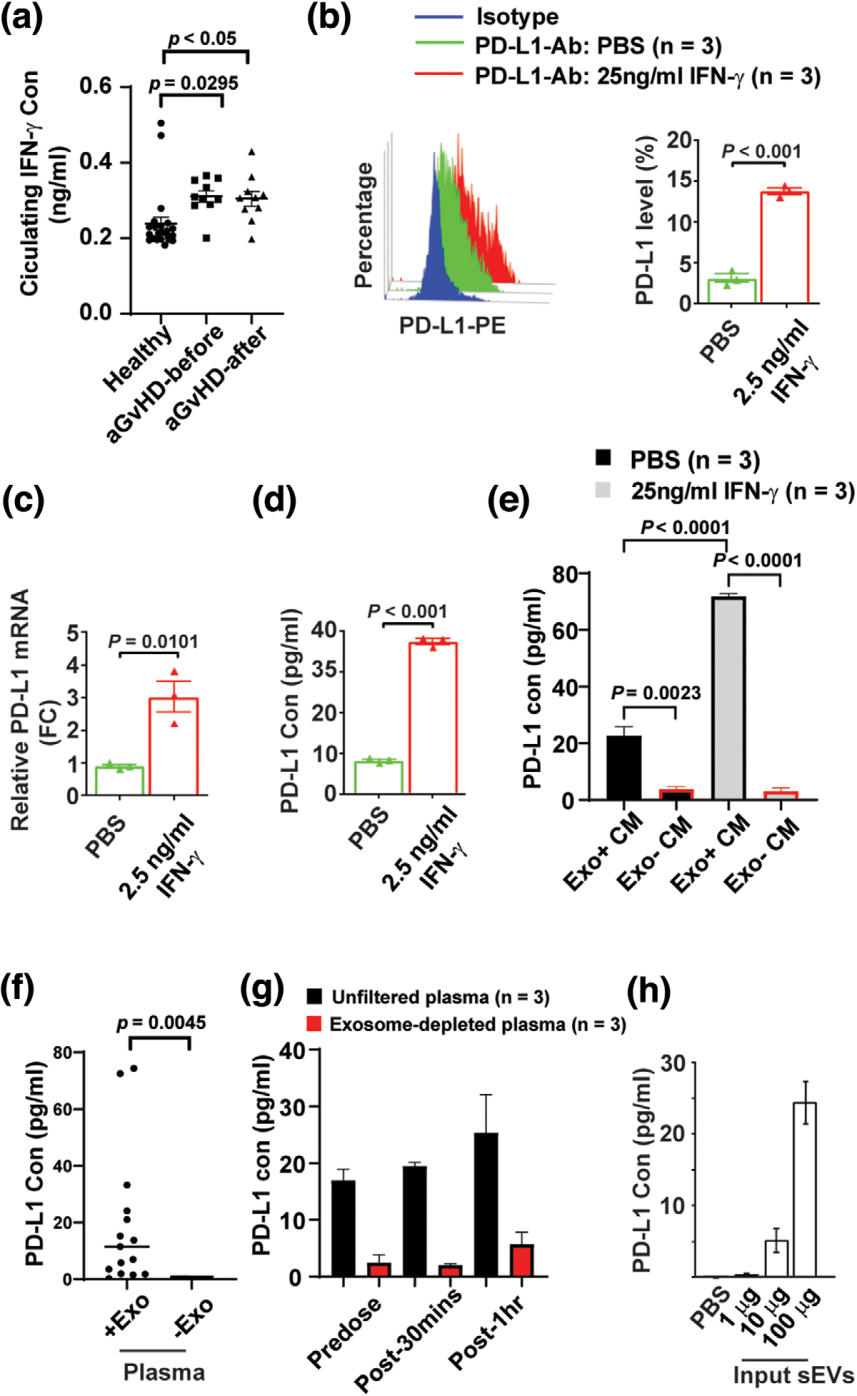
IFN‐γ induces the sEV‐PD‐L1 release from WJMSCs. (a) Quantitation of IFN‐γ in the plasma samples of healthy donors (*n* = 23) and aGvHD patients (*n* = 10) before or after WJMSC infusion, measured by ELISA. (b) Flow cytometry of PD‐L1 protein on the WJMSCs induced by 2.5 ng/ml IFN‐γ (left) and quantitative analysis (right, *n* = 3). (c) Quantitation of PD‐L1 mRNA in the WJMSCs from b (*n* = 3). (d), ELISA of PD‐L1 secreted from the WJMSC from b (*n* = 3). (e) PD‐L1 in both complete conditioned medium (Exo+ CM) and exosome‐depleted conditional medium (Exo‐ CM) from WJMSCs licensed by IFN‐γ (*n* = 3). (f,g) Quantitation of PD‐L1 in unfiltered or exosome‐depleted human plasma samples. Exosome‐free human plasma was generated by either ultracentrifugation (F, *n* = 3) or Amicon Ultra‐4 centrifugal filters (*n* = 3). (h) ELISA of PD‐L1 on WJMSC‐derived sEVs (*n* = 15). Data are mean ± s.e.m. and analysed by unpaired one‐tailed Student's t‐test (a‐h)

Interferon‐γ (IFN‐γ) can promote MSCs to secrete soluble PD‐L1 (Carvalho et al., 2019; Davies et al., 2017; Francisco et al., 2009; Guan et al., 2018; Krampera, 2011) and enhance the release of exosome‐associated PD‐L1 in other cell types (Chen et al., 2018; Ricklefs et al., 2018). To explore whether increased circulating sEV‐PD‐L1 was related to patient plasma IFN‐γ levels, we first examined the levels of plasma IFN‐γ in aGvHD patients. We observed that the systemic IFN‐γ concentration in aGvHD patients was significantly higher (increased by 69%, *P *< 0.005) compared with healthy individuals (Figure 7a). In addition, we noticed that WJMSC infusion did not affect patients’ plasma IFN‐γ levels. Second, consistent with previous findings (Carvalho et al., 2019; Davies et al., 2017), we observed that IFN‐γ significantly up‐regulated expression of cell surface protein (Figure 7b) and mRNA (Figure 7c) of PD‐L1 in WJMSCs in vitro. Consistent with these observations, we found that PD‐L1 concentration increased by 4.5‐fold (*n* = 3, *P *< 0.001) in the culture medium from IFN‐γ‐treated WJMSCs (Figure 7d). To determine whether IFN‐γ can potential induced soluble PD‐L1 from WJMSC infusions, we examined the level of PD‐L1 protein in complete/unfiltered (Exo^+^) conditioned medium and exosome‐depleted (Exo^–^) conditioned medium (Figure 7e). Approximately 3.8 ± 1.56 ng/ml PD‐L1 in the untreated culture and 3.1 ± 2.0 ng/ml PD‐L1 in the IFN‐γ‐treated culture was detected in exosome‐free conditional medium. This finding suggests that IFN‐γ cannot directly enhance WJMSCs to secrete soluble PD‐L1. 

Third, we sought to determine the fraction of membrane‐bound PDL1 carried by the sEVs to total PDL1 protein in aGvHD patients’ plasma post WJMSCs infusion. To address this question, we compared the levels of PD‐L1 in the total versus EV‐depleted plasma samples. About 19 ± 6 pg/ml PD‐L1 (*P* = 0.0045) was detected in the unfiltered plasma, but only 0.02 ± 0.009 pg/ml in the exosome‐free plasma generated by Ultracentrifugation (Figure 7f). Consistently, significantly lower levels of PD‐L1 were detected in exosome‐depleted plasma compared to complete/unfiltered plasma (Figure 7g). These findings suggest that a majority of circulating PD‐L1 in the plasma was sEV membrane‐bound and was not soluble. Indeed, WJMSC sEV‐PD‐L1 were able to be directly detected by ELISA (Figure 7h). Our results indicated that exposing WJMSCs to the pro‐inflammatory cytokine IFN‐γ may be an initial step to induce the release of immunosuppressive sEV‐PD‐L1 after WJMSC infusion in vivo.

### 2.8 Binding of WJMSC sEVs to the peripheral blood cells in vivo

**FIGURE 8:**
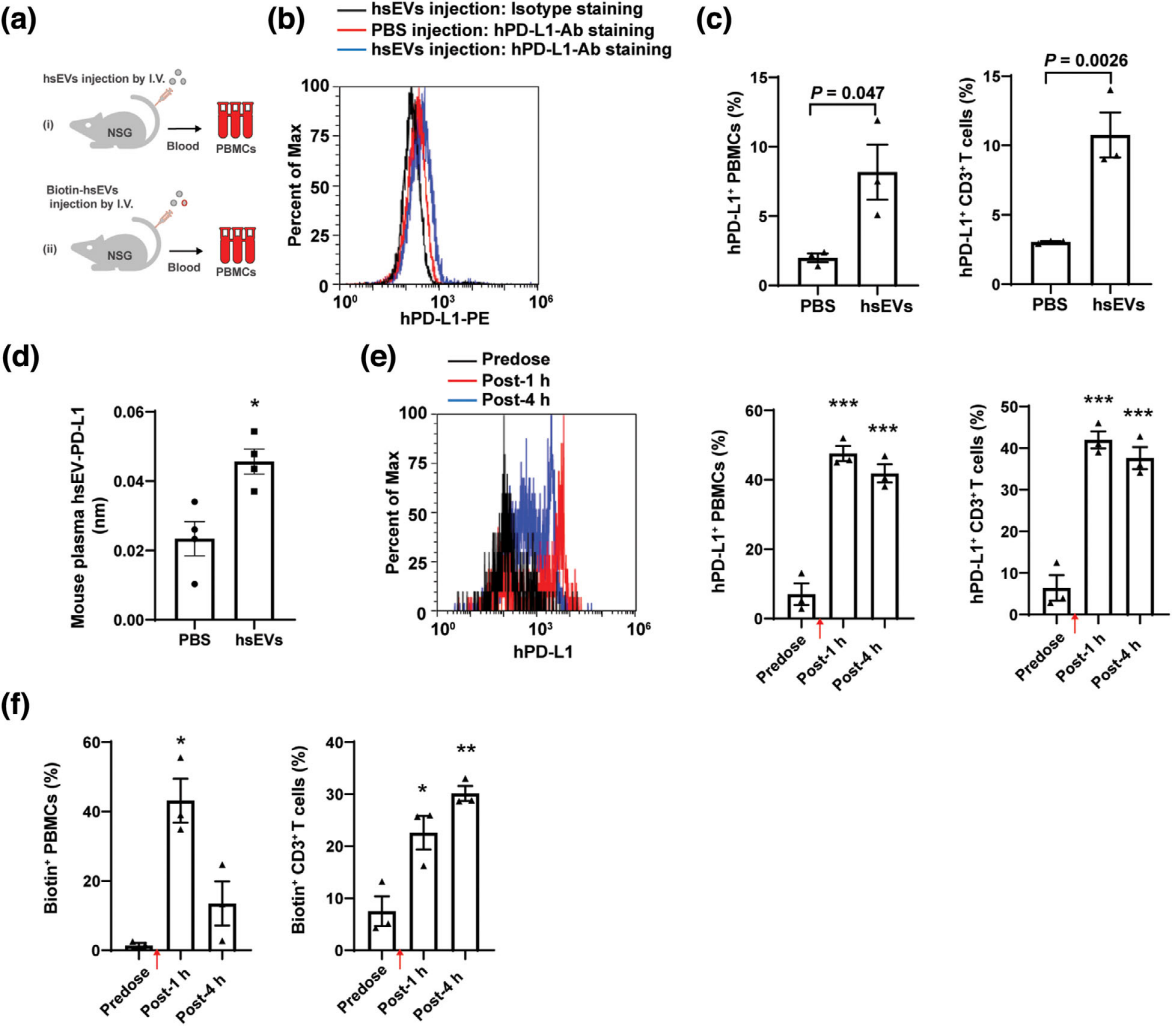
In vivo binding of human WJMSC sEVs on mouse PBMCs. (a) Schematic of intravenously administrating 4 mg/kg sEVs (**i**) or Biotin‐labelled sEVs (**ii**) to immunodeficient NOD scid gamma (NSG) mice. (b) Flow cytometry of human PD‐L1 on the cell surface of PBMCs from NSG mice administrated by PBS or sEVs for 12 h. (c) Quantitation of human PD‐L1 on mouse PBMCs (left, *n* = 3) and CD3+ T cells (right, *n* = 3) from b. (d) Quantitative analysis of human sEV‐PD‐L1 in mouse plasma samples from b (*n* = 4). (e) Flow cytometry showing human PD‐L1 on mouse PBMCs from NSG mice before and after WJMSC sEV injection (left) and quantitation (right, *n* = 3). (f) Quantitative analysis of biotin labelled WJMSC sEVs (Biotin^+^) on mouse PBMCs (left, *n* = 3) and CD3^+^ T cells (right, *n* = 3), measured by flow cytometry. Human WJMSC sEVs infusion time points were indicated by red arrows (d,e). Data are mean ± s.e.m. and analysed by unpaired one‐tailed Student's t‐test (c‐f). ^*^ *P *< 0.05, ^**^ *P *< 0.01, ^***^ *P *< 0.005

A decline in circulating sEV‐PD‐L1 at later timepoints was observed in patients (Figure 5a). IFN‐γ remained constant in aGvHD patients after WJMSC infusion (Figure 7a), and one possibility might be the direct uptake of sEVs by peripheral blood cells. To address this possibility, we intravenously administrated WJMSC sEVs to immunodeficient NOD SCID gamma (NSG) mice (Figure 8a,i) and measured the presence of human PD‐L1 (hPD‐L1) on the surface of mouse peripheral blood cells by flow cytometry (Figure 8b). Twelve hours after WJMSC sEV injection, about 8.2 ± 2.0% of the mouse PBMCs and 10.8 ± 1.6% of the mouse CD3^+^ T cells appeared positive for hPD‐L1 (Figure 8c). Even though an unavoidable low (background) level of 2.0 ± 0.31% PBMCs and 3.0 ± 0.06% CD3^+^ T cells was detected, our results suggested that binding and subsequent uptake of human sEVs by mouse blood cells might be a major reason why hPD‐L1 was detectable on mouse cells. We further measured the levels of human PD‐L1 on the isolated sEVs from mouse plasma samples by BLI. For WJMSC sEV‐injected mice, we found a two‐fold increase (*P *= 0.011) of human PD‐L1 on isolated mouse sEVs compared with PBS‐injected mice (Figure 8d). Within 1–4 h after sEV injection, a higher percentage of hPD‐L1‐positive mouse PBMCs (42 ± 2.7%∼48 ± 2.2%) and CD3^+^ T cells (38 ± 2.6%∼42 ± 2.01%) were detected (Figure 8e). To confirm this uptake, biotin‐labelled WJMSC sEVs were injected into NSG mice (Figure 8a,ii) and similar elevated levels of biotin positive PBMCs (13 ± 6.3%∼43 ± 6.3%) and CD3^+^ T cells (22.6 ± 3.2%∼30 ± 1.4%) were observed (Figure 8f). In addition to inflammatory environment exposure, our results suggest that direct uptake of sEVs by peripheral blood cells may likely influence circulating sEV‐PD‐L1 levels.

## 3 DISCUSSION

Steroid refractory aGvHD is a major cause of mortality in patients receiving HCT. While the immunomodulatory characteristics of MSCs represent a potential effective therapy with minimal toxicity, the exact mechanisms for their therapeutic function in vivo have been elusive. Gaining insight into the specific contribution of sEVs to WJMSCs clinical effects provides opportunities for enhanced cellular‐based therapeutics in patients with aGvHD. We demonstrate for the first time that WJMSCs secrete immunosuppressive sEVs after being infused into aGvHD patients and subsequently target the immune cells via enhancing T cell suppression. We identified PD‐L1, an inhibitory checkpoint protein expressed on MSCs (Carvalho et al., 2019; Krampera, 2011) and involved in their immunomodulation (Davies et al., 2017; Francisco et al., 2009; Guan et al., 2018), as specifically enriched in WJMSC‐derived sEVs. Our study reveals a role of sEV‐carried PD‐L1 for WJMSC‐based therapy in aGvHD patients.

A role for MSC‐derived sEVs in T cell regulation has been suggested in various settings (Fujii et al., 2018; Ha et al., 2020; Lai et al., 2018; Zhang et al., 2018; Zhang et al., 2018), mainly in the production and differentiation of T helper cells (Fujii et al., 2018; Lai et al., 2018) and regulatory T cells (Lai et al., 2018; Zhang et al., 2018; Zhang et al., 2018). We report that the inhibitory PDL1 checkpoint is enriched on WJMSC‐derived sEVs, thus suggesting these sEVs might regulate the functions of T cell receptors (TCR) responsible for recognizing antigen peptides. Indeed, we show that WJMSC sEVs effectively inhibit both CD4^+^ and CD8^+^ T cell activation that is mediated by TCR signalling pathway. sEVs only demonstrate their immunosuppressive capability without stimulating the activation or enhancing the proliferation of naïve T cells. Consistent to previous studies (Chen et al., 2016; Fujii et al., 2018), our studies support that sEVs’ functions, exerted on the TCRs, primarily rely on indirectly modulatory regulation rather than the direct antigen presentation.

We show that PD1, the receptor for PD‐L1, is up‐regulated on the activated CD4^+^ T cells, underscoring the roles of PD‐L1‐PD1 axis in sEV‐related immunomodulation. Blockage of PD‐L1 on the WJMSC sEVs through neutralizing antibodies impairs their inhibitory ability for TCA. Consistently, PD‐L1‐deficient sEVs from PD‐L1 null WJMSCs failed to block the TCA, supporting that sEVs inhibit T cell activation via sEV‐carried checkpoint PD‐L1. Furthermore, sEV‐PD‐L1 is essential for the phosphorylation of pZAP70, an important partner protein associated with the activated TCR and its subsequent induction of downstream signalling pathways. Our study suggests that WJMSC‐derived sEVs inhibit T cell activation or enhance T cell suppression through membrane‐carried inhibitory checkpoints.

While MSC therapy faces challenges from either resources or the standard definition of MSCs (Brown et al., 2019; Galipeau & Sensébé, 2018), sEVs have significant potential as a novel alternative to whole cell therapies because of the low toxicity and ease of storage (Galipeau & Sensébé, 2018). In aGvHD patient with WJMSC infusion, we demonstrate that a rapidly increasing plasma sEV‐PD‐L1 following infusion. Exposing WJMSCs to IFN‐γ seems critical to release sEV‐PD‐L1 from their parental cells. Secreted sEV‐PD‐L1 can quickly target the peripheral blood cells through direct uptake of sEVs. Remarkably, we show that increased sEV‐PD‐L1 in patients’ plasma samples is associated with improved GvHD after clinical WJMSC infusion, suggesting that sEV‐PD‐L1 is an important mechanism explaining the efficacy of WJMSC in aGvHD patients.

**FIGURE 9:**
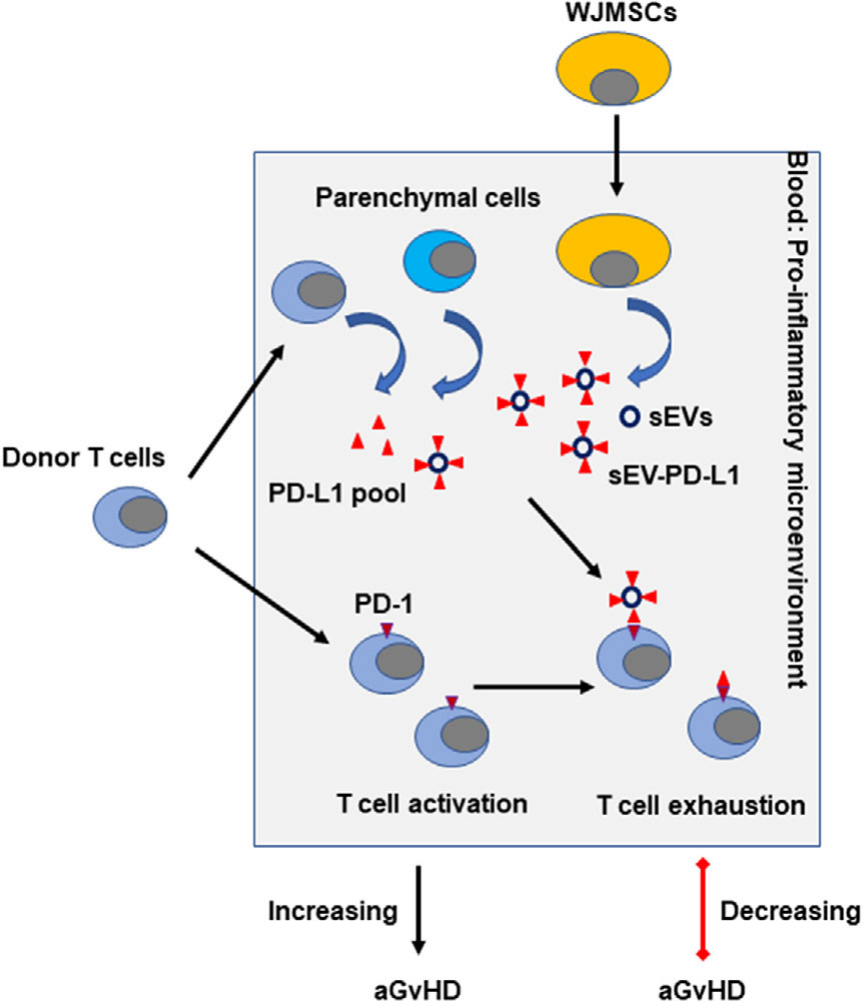
Summary of WJMSCs’ contribution to circulation PD‐L1 pool through secreting sEVs in aGvHD patients. Transplanted WJMSCs are induced by IFN‐γ and enhance the release of sEV‐carried PD‐L1 in the blood. Membrane‐bound sEV‐PD‐L1 interacts the PD1 on the T cells and modulate the TCR‐mediated TCA associated with aGvHD

Taken together, our findings suggest that infused WJMSCs can significantly contribute to the circulation “PD‐L1 pool” by quickly secreting sEVs (Figure 9) and may represent the fundamental mechanism underlying WJMSC‐based therapies. We also showed that sEV‐associated PD‐L1, rather than its soluble form, mainly contributes to the inducible “PD‐L1 pool” though endogenous soluble PD‐L1 is detected in patients. A more detailed characterization of the effects of sEV‐PD‐L1 on T cells should shed light on the contribution of therapeutic exosomes to T cell regulation.

Tumour cell‐derived exosomes carrying PD‐L1 have been suggested to contribute to malignant immunosuppression, but are often associated with the clinical benefits of compensatory immune checkpoint targeted therapies (Chen et al., 2018; Poggio et al., 2019; Ricklefs et al., 2018). Although tumoral exosome‐PD‐L1 has been reported to be deleterious for cancer patients (Chen et al., 2018; Poggio et al., 2019; Ricklefs et al., 2018), our findings indicate that WJMSC sEV‐PD‐L1 might indeed be beneficial for treating aGvHD patients through targeting CD4^+^ T cells. However, increased sEV‐PD‐L1 may enhance both inhibitory PD‐L1/PD1 (Wolfgang Koestner et al., 2011) and stimulatory PD‐L1/CD80 (Cassady et al., 2018; Ni et al., 2017) on the donor CD8^+^ T cells related to the GvL. Regarding the former point, the completed clinical trial (NCT03158896) of patients with hemopoietic malignancies have evaluated malignancy recurrence and did not show increased malignancy relapse post‐WJMSC therapy even with longer follow up (Soder et al., 2020). It has been suggested that MSC‐associated exosomes may reduce tumour growth by impairing angiogenesis and increasing apoptosis (Brossa et al., 2020). The identification of sEV‐carried PD‐L1 further expands the potential application of WJMSC‐derived sEVs for their anti‐tumour therapy through PD‐L1/CD80 checkpoint regulation. More importantly, regarding their immunomodulation roles (Gomzikova et al., 2019), our study offers, for the first time, a rationale to further develop a new generation of cell free therapeutics for aGvHD based on WJMSC‐derived sEVs.

## 4 METHODS

### 4.1 Production and maintenance of therapeutic WJMSCs

Umbilical cord specimens were obtained from individuals enrolled under the Institutional Review Board‐approved protocol (HSC#1546) following good manufacturing practices (GMP). Umbilical cords were collected from healthy women (18∼35‐year old) who underwent elective cesarean section after full‐term pregnancy. The cord was thoroughly washed twice with sterile phosphate buffered saline (PBS), blood vessels were removed, and pieces of cord tissue (approximately 2–3 mm in diameter) were seeded onto tissue culture dishes containing Stem MACS MSC Expansion Media (Miltenyi Biotec, Germany), penicillin 100 U/ml, and streptomycin 100 µg/ml (Mediatech). Explant cultures were incubated at 37°C in a humidified atmosphere containing 5% CO_2_.Medium was changed every 3–4 days and tissue explants were removed after 21 days of culture to allow the migration of cells from the explants. Once between 80–90% confluency, adherent cells were trypsinized using TrypLE Select (Life Technologies) and reseeded in tissue culture flasks (Corning) for further culture expansion. Low‐passage WJMSCs (< 4 frozen/thawed cycles) were cultured in Stem MACS MSC Expansion Media. High‐passage WJMSCs (> 4 < 10 frozen/thawed cycles) were cultured in conditional DMEM media supplied with 15% (v/v) fetal bovine serum (FBS).

### 4.2 In vitro WJMSC differentiation

WJMSCs were differentiated into fat cells, cartilage cells and bone cells using human MSC differentiation kits following the standard protocol provided by the manufacturer (ThermoFisher Scientific, USA). Differentiated cells were fixed with 4% formaldehyde (PFA) solution and washed twice with PBS for histochemical analysis. In brief, bone cells were stained with 2% Alizarin Red S (Sigma, USA) prepared in H_2_O for at least 45 min, cartilage cells were stained with 1% Alcian blue (Millipore, USA) solution prepared in 0.1 N HCL for 30 min and fat cells were stained with 0.5% Oil red O (Sigma, USA) solution prepared in propylene glycol for 50–60 min. Images were recorded by using Nikon Digital Imaging Head (Nikon, Japan) and analysed by MetaMorph 7.7.0 imaging software (Molecular Devices, USA).

### 4.3 Flow cytometry

WJMSCs were characterized by flow cytometry using the BD Stemflow Human MSC analysis kit (BD Biosciences, USA). WJMSCs were washed with PBS and detached using TrypLE Select and resuspended in staining buffer (1% FBS in PBS). WJ‐MSCs were stained with multiple fluorochrome‐conjugated antibody cocktails for positive and negative selection markers. Positive marker cocktail included APC‐conjugated anti‐CD73, FITC‐conjugated anti‐CD90, and PerCP‐Cy5.5‐conjugated anti‐CD105. The negative marker cocktail included PE‐conjugated antibodies against CD45, CD34, CD11b, CD19, and HLA‐DR. WJMSCs were stained with PE‐conjugated anti‐human PD‐L1 (Biolegend, USA). Human PBMCs were stained by anti‐human antibodies listed as below: Anti‐ CD3 PE‐Cy5/CD4 PE/CD8 FITC Cocktail, APC anti‐ CD154 and PE‐dazzle anti‐ ZAP70 Phospho (Tyr319)/Syk(Tyr352), and APC/fire anti‐PD1 antibodies were purchased from Biolegend (USA). Antibodies anti‐ CD3, CD4 APC, CD8 FITC, CD14PerCP, and IFN‐γ PE proteins were purchased from Miltenyi Biotec (Germany). To stain anti‐IFN‐γ and pZAP7 antibodies, cells were fixed with 4% formaldehyde and permeabilized with 10 × permeabilization buffer (Invitrogen, USA). Human sEVs on mouse PBMCs were stained by anti‐human PD‐L1 PE (Biolegend, USA) and rat anti‐mouse CD3 FITC (Biolegend, USA). Biotin‐labelled human sEVs on mouse PBMCs were stained by streptavidin conjugated with PE/Cyanine 7 (Biolegend, USA) and rat anti‐mouse CD3 FITC (Biolegend, USA). All antibodies were used at a dilution of 1:100. Flow cytometric analysis was performed using BD LSR II analyser (Becton Dickinson, USA) or Attune NxT multiparameter flow cytometer (Invitrogen, USA).

### 4.4 sEV enrichment and characterization

Either 500 ml supernatant from WJMSC culture or 1 ml blood plasma sample from aGvHD patients was spun down at 400 x *g* for 10 min to remove cell debris. Then, the supernatant was spun down at 2000 x *g* for 30 min to remove apoptotic bodies. This was followed by ultracentrifugation spins at 10,000 x *g* for 1.5 h and 100,000 x *g* centrifugation for 1.5 h, both at 4°C. The pellet was washed with PBS and spun 100,000 x *g* centrifugation for 1.5 h at 4°C. Finally, the pellet was resuspended in PBS. To enrich for small EVs, extracellular vesicle pellets were further passed through qEVoriginal/70 nm columns (Izon Science, USA) according to the protocol provided by the manufacturer. The size of the sEVs was measured by NanoSight LM10 system and data was analysed using the NTA software v2.3 (NanoSight Ltd; United Kingdom).

### 4.5 Immunoblotting

WJMSCs and sEVs were lysed with RIPA buffer containing a Halt protease Inhibitor single‐use cocktail (Thermo Scientific). Membrane‐bound PD‐L1 were extracted using Mem‐PER™ plus membrane protein extraction kit following the manufacturer's instruction (Thermoscientific, USA). Protein lysates were separated by a Mini‐protean TGX precast gel (BIO‐RAD, USA) and transferred onto a PVDF membrane (BIO‐RAD, USA). Western blots were performed according to the standard techniques. The following antibodies were used at a dilution of 1:1000 in 5% nonfat milk unless otherwise stated: goat anti‐human PD‐L1 and PDL2 (R&D), Rabbit anti‐human CD90, CD105 (Thermo Scientific), Rabbit anti‐human CD9, CD81, HSP70, and CD63 (System Biosciences) and mouse anti‐human β‐actin (1:10,000, Sigma). Secondary antibodies include donkey anti‐goat‐HRP (R&D), goat‐anti‐rabbit‐HRP (Cell Signalling) and goat anti‐mouse antibody conjugated with HRP (Sigma). Signals were developed by using Pierce ECL Western Blotting Substrate (Thermo Scientific).

### 4.6 Transmission Electron Microscopy (TEM)

TEM was carried out as previously reported (Chen et al., 2018). In brief, 10 µl WJMSC sEVs were dropped on the spot plate and glow discharge carbon filmed nickel grids (Electron Microscopy Sciences, USA) were floated on the drops for 20 min. Grids were washed three times with H_2_O and fixed with 2.5 glutaraldehyde in 100 mM sodium cacodylate buffer (PH 7.0) for 1 h. Negative staining was performed by using 3% solution of neutral sodium phosphotungstate for 20 s. For immune‐gold staining, grids were incubated with mouse monoclonal Leaf™ purified anti‐human PD‐L1 antibody (1:250, Biolegend, USA) for 60 min. Grids were washed three times with H_2_O followed by incubation with goat anti‐mouse IgG conjugated with gold (1:500, Abcam, USA) for 1 h. Again, grids were then washed three times with H_2_O. Finally, grids were fixed with 1% glutaraldehyde and stained with 1% uranyl acetate for 5 s, dried and viewed under a JEOL JEM‐1400 transmission electron microscope (JEOL, USA) equipped with a Lab6 gun at 100 KV.

### 4.7 Enzyme‐linked immunosorbent assay (ELISA)

Samples from blood plasma, cell culture supernatant and purified WJMSC sEVs were added to 96‐wll plate to detect the human PD‐L1 using a Human/Cynomolgus Monkey B7‐H1 Elisa kit according to the manufacturer's instructions (R&D systems, USA). For detection of IFN‐γ, ST2 and REG3 alpha (REG3A), plates were prepared using Duoset human IFN‐γ, ST2 and REG3A ELISA kit according to the manufacturers’ instructions (R&D systems, USA). Coated plates were added by the diluted plasma samples from aGvHD patients and detected by ELISA detection antibodies (R&D systems, USA). O.D. Value was read at an absorbance of 450 nm using Infinite 200 PRO plate reader (Tecan US). To determine soluble PD‐L1 in conditional medium and human plasma samples, exosome‐free conditional medium or plasma samples were generated by either ultracentrifugation (shown as above) or Amicon Ultra‐4 centrifugal filters according to the manufacturer's instructions (Millipore, USA).

### 4.8 Quantitative PCR (qPCR)

The mRNA expression of human PD‐L1 was examined by qPCRs. Forward primer: 5′‐GCAAGGCGATTAAGTTGGGT‐3′ and Reverse primer: 5′‐GTGCTCTTCATCTTGTTGGT‐3′ were designed. Total RNA was extracted from cells by using TRIzol reagent (ambio, USA). The first‐strand cDNA was synthesized using High Capacity cDNA Reverse Transcription Kit (Applied biosystems, USA) according to the manufacture's protocols. Quantitative PCRs were conducted following the standard protocols using Maxima SYBR Green/Rox qPCR Master Mix (2X) (Thermo scientific, USA). Reactions were conducted on CFX96 Real‐Time System (Bio‐Rad, USA) following the standard procedures. qPCR experiments were independently repeated three times.

### 4.9 Immunofluorescence staining

Human PBMCs were isolated with Ficoll‐Hypaque solution (STEMCELL, USA) by centrifugation at 400 x *g* for 30 min followed by washing with PBS. CD3^+^ T cells were further sorted using CD3 micro beads (Miltenyi Biotec, Germany). Sorted CD3^+^ T cells were activated by CD3/CD28 Dynabeads (1:1 ratio) and seeded on WJMSCs. PBMCs and WJMSCs were cocultured in conditional DMEM medium supplied with 10% FBS for overnight. Immunofluorescence staining was carried out by following the standard procedures. Cells were fixed with 4% PFA and stained by rabbit anti‐human CD3 (1:250, Cell Marque, USA) and mouse anti‐human PD1 (1:250, eBioscience, USA) for 1 h. These were then washed thrice with PBS. Then, the cells were incubated with goat secondary antibodies anti‐mouse IgG‐ Alex594 (1:500, Invitrogen, USA) and anti‐rabbit IgG‐FITC (1:500, Invitrogen, USA). For PD‐L1 staining, WJMSCs were cultured in conditioned DMEM medium supplied with 10% FBS and induced with 2.5 ng/ml human recombinant IFN‐γ protein (R&D, USA). Cells were stained with rabbit anti‐human PD‐L1 antibody (1:250, Thermal scientific, USA) and washed thrice with PBS. These cells were stained with anti‐rabbit IgG conjugated with Alex 594. Finally, cells were mounted with VECTASHIELD mounting medium with DAPI (Vector, USA). Images were recorded by using Nikon Digital Imaging Head (Nikon, Japan) and analysed by MetaMorph 7.7.0 imaging software (Molecular Devices, USA).

### 4.10 Clinical blood sample preparation

The Midwest Stem Cell Therapy Center at the University of Kansas Medical Center has developed and manufactured clinical grade WJMSCs (MSCTC‐0010) for use in humans. Therapeutic WJMSCs have been tested in a Phase I clinical trial in subjects with high‐risk aGvHD and this study showed preliminary safety of two doses of therapeutic WJMSCs by IV administration (ClinicalTrials.gov Identifier: NCT03158896 – “Evaluation of Umbilical Cord‐Derived Wharton's Jelly Stem Cells for the Treatment of Acute Graft Versus Host Disease”). A total of 10 subjects were treated in two dose cohorts of five subjects; five patients had high risk acute myelogenous leukaemia, two myelodysplastic syndrome, two myelofibrosis, and on1 had T cell non‐Hodgkin's lymphoma. The study was approved by the institutional review board and was conducted in accordance with the principles of the Declaration of Helsinki and International Conference on Harmonization Good Clinical Practice Guidelines. All the patients provided written informed consent (additional details regarding clinical trial design and outcomes are in Soder et al., 2020 (Soder et al., 2020)). Cells were administered via IV infusion at a dose of 1.2 × 10^6^ WJMSCs/kg for two doses in the first cohort and 10 × 10^6^ WJMSCs/kg in the second cohort and the subjects were followed over a six‐month period. Patient blood samples were collected prior to the first dose of and then weekly for 4 weeks and patients’ aGvHD grades were further evaluated according to the clinic diagnostic standards. For dynamic analysis, patient blood samples were collected at individual timepoints before WJMSCs administration (pre‐dose) and after WJMSCs administration (post‐30 min, post‐1 h, post‐2 h, post‐4 h, and post‐8 h).

### 4.11 ExoView

sEV numbers in blood plasma were measured using ExoView™ Tetraspanin kits (NanoView Bioscience, USA) according to manufacturer's instructions. In brief, human and mouse plasma samples were diluted with incubation solution (1:1 dilution). 35 µl of diluted plasma samples was loaded on the Nanoview chips and incubated overnight. Each chip included three mouse capture antibodies targeted against sEV tetraspanin markers (CD9, CD63, CD81). After washing thrice with incubation solution, Nanoview chips were stained with detection antibodies cocktail for 1 h. Detection antibody cocktail contained three mouse antibodies anti‐human CD81 conjugated with Alex 488, CD63 conjugated with Alex 647 and CD9 conjugated with Alex 555. Again, these were washed thrice with incubation solution, immunofluorescent staining on the chips was recorded with ExoView™ R100 automated imager and analysed using ExoScan 2.5.5 acquisition software (NanoView Bioscience, USA).

### 4.12 In vivo WJMSC sEV injection and blood collection

All animal experiments were performed according to protocols (2017‐2387 & 2020–2549) approved by the Institutional Animal Care and Use Committee (IACUC) of the University of Kansas Medical Center. 4 mg/kg human WJMSC sEVs were injected into 8‐week immunodeficient NSG mice (NOD.Cg‐*Prkdcscid Il2rgtm1Wjl/SzJ*) through intravenous (IV) injection according to a previous protocol (Ehx et al., 2018). WJMSC sEVs were labelled with biotin using EZLabel™ antibody biotin labelling kit according to the standard protocol provided by the manufacturer (Biovision, USA). 4 mg/kg biotin labelled WJMSC sEVs were injected into mice through intravenous (IV) injection. 1 h, 4 h, or 12 h after injection, mouse facial blood withdrawal was performed using 5 mm animal lancet (Goldenrod, USA) and about 2–3 drops of blood samples were collected into an EDTA micro‐collection tube (Microtainer, USA). To obtain PBMCs, whole mouse blood was diluted with RBC lysing solution (Biolegend, USA) and kept at RT for 10 min. Cells were spun for 10 min at 250 x *g* and washed with PBS for one time.

### 4.13 In vitro TCR‐mediated T cell activation

All patients who provided blood samples for this study did so under written informed consent and University of Kansas Medical Center Institutional Review Board approved the collection protocol (HSC #5929) and following U.S. Common Rule. De‐identified clinical samples were provided by the KU Cancer Center's Biospecimen Repository Core Facility (BRCF) along with detailed clinical outcomes and phenotypes. Once the patient provides written, informed consent in accordance with the BRCF IRB protocol, a blood sample is collected, the sample is de‐identified to the user, and transported directly to the laboratory for processing. Five ml whole blood was used to prepare PBMCs using lymphocyte separation medium (Corning, USA) and spun for 30 min at 400 x *g* through density gradient centrifugation. After washing with PBS, 1 × 10^5^ PBMCs per well were seeded in 96‐well plates with 1 ml of Hyclone™ RPMI‐1640 medium (GE healthcare life sciences, USA) supplemented with 10% FBS, 10 µM HEPES buffer, 100 U/ml penicillin‐streptomycin. PBMCs were activated with CD3/CD28 Dynabeads (Gibco, USA) at a dilution of 1:1 ratio as previously described (Chattopadhyay et al., 2005; Chattopadhyay et al., 2006). T cell activation was measure by dual CD154^+^/CD4^+^ or PD1^+^/CD4^+^ T cells with flow cytometry. Unstimulated or stimulated PBMCs were treated with 0.2‐40 µg/ml WJMSC sEVs. Stimulated PBMCs were treated with 2.5‐40 ng/ml human recombinant PD‐L1 protein (R&D systems, USA) for 12 h. Stimulated PBMCs were treated with 10 µg/ml WJMSC sEVs, 3 µg/ml anti‐human PD‐L1 internalization antibody/IgG (Novus biologicals, USA), or 1 µg/ml recombinant human PD‐L1 protein (R&D systems, USA) for overnight. Stimulated PBMCs were treated with WT (*PD‐L1^+/+^*) or KO (*PD‐L1^–/–^*) WJMSCs for 48 h and the ratio of WJMSCs to PBMCs were 1 to 10. Stimulated PBMCs were treated with WT (*PD‐L1^+/+^*) or KO (*PD‐L1^–/–^*) WJMSC‐derived sEVs for both 12 h and 36 h. For CD8^+^ T cell activation, PBMCs were isolated from donors and stimulated with 1 ng/ml PepTivator CMV‐pp65 peptide (Miltenyi Biotec, Germany). Intracellular IFN‐γ in CD8^+^ T cells was examined by using Rapid Cytokine Inspector kit (Miltenyi Biotec, Germany) following the manufacturer's instruction.

### 4.14 Biolayer interferometry (BLI)

All solutions were made in octet kinetics buffer (1x PBS with 0.1% BSA and 0.02% Tween‐20). Goat anti‐human PD‐L1 antibody or IgG control (R&D systems, USA) was diluted in 200 µl kinetics buffer with the final 5 ng/ml concentration. 20 µg WJMSC‐derived sEVs and 0–1 mg/ml human recombinant PD‐L1 protein (R&D systems, USA) were suspended in 200 µl in kinetics buffer. Each kinetic experiment consisted of four steps: (1) protein G biosensors were equilibrated in kinetics buffer for 20 min, (2) goat anti‐human PD‐L1 antibody/IgG control were loaded onto the protein G biosensors for 400 s (3) a baseline was established in kinetics buffer for 1 min, (4) binding WJMSC‐derived sEVs 1–2k s. BLI experiments were recorded in sterile PBS at 25°C on Octet Red instrument (ForteBio LLC, USA). Kinetic assay was performed at 25°C with 1000 rpm plate rotation. Software provided with the Octet system (version 7.1) was used to fit the data to a one‐to‐one model and obtain *k_on_*, *k_off_*, and *K_D_* values.

### 4.15 PD‐L1 Knockout (KO) in WJMSCs and characterization

*PD‐L1* gene in WJMSCs was genetically disrupted by CRIPR‐cas9 gene editing as described in the previous studies (Cong et al., 2013; Shalem et al., 2014). *Pdl1*sgRNA (targeting uCUG): 5′‐GGGCCAGTCTCCTCGCCTGC‐3′ and *Pdl1*sgRNA (targeting uAUG): 5′‐TGGTCCCCAAGCCTCATGCC‐3′ were designed and by Shalem et al (Shalem et al., 2014) and cloned into pSpCas9(BB)‐2A‐GFP (PX458) vector (Addgene no. 48138). Non‐targeting control sgRNA was used side‐by‐side with *PD‐L1* sgRNA to rule out phenotypes result from nonspecific editing. HEK293T cells (Addgene) were co‐transfected with packaging vectors psPAX2 and pCI‐VSVG (Addgene) with above lentiviral vectors using lipofectamine 2000 (Invitrogen). Media of the HEK293T cells was changed 18 h after transfection and viral supernatants were collected 24 and 48 h later and filtered to transfect WJMSCs. After infection, WJMSCs were cultured in DMEM medium supplied 10% FBS and positive clones were screened by adding 0.5‐2 µg/ml puromycin. To confirm *PD‐L1* gene knockout, genomic DNAs were isolated from WJMSCs using QIAamp DNA (Qiagen, USA). A 300 nt DNA fragment covered the editing target region was amplified by PCR and cloned into pCRII vector using TA cloning kit (Thermal fisher, USA). The T7 and Sp6 promoters provide on the pCR II vector were used to sequence cloned DNA fragments. Sequence alignment was performed using bioinformatics software DNASTAR (Dnastar, USA).

### 4.16 PD1/NFAT assay

PD1/NFAT reporter Jurkat T cells were purchased from BPS Bioscience (USA) and cultured with growth medium 2A conditional medium supplied with 10% FBS. Cells were activated by human T‐activator CD3/CD28 Dyna beads (1:1 ratio, Gibco, USA). Activated cells were treated with 20 µg/ml PD‐L1 WT or KO WJMSC EVs, 3 µg/ml PD‐L1 antibody or isotype control and 1 µg/ml recombinant human PD‐L1 protein. Luciferase assay was conducted using One‐Step Luciferase assay system (BPS Bioscience, USA) according to the procedures provided by the manufacturer. O.D. Value was read using Infinite 200 PRO plate reader (Tecan, US).

### 4.17 MTT assay

CD3^+^ T cells were sorted from healthy human PBSMCs using CD3 micro beads (Miltenyi Biotec, Germany). CD3^+^ T cells were seeded into 96‐well plates in RPMI‐1640 medium supplied with 10% FBS and treated with 10 µg/ml WJMSC sEVs for 6 days. Cell toxicity was examined by using MTT assay kit (BioVision, USA) according to the manufacturer's instructions. Plates were read by a micro‐plate reader at 490 nm wavelength as above.

### 4.18 Statistics

Data were analysed and statistics performed in GraphPad Prism 8. Data were expressed as means ± s.e.m (standard error of mean). A one‐tailed, unpaired Student's *t* test was used for pair‐wise comparison or one‐way analysis of variance (ANOVA) with Dunnett's test. For correlation analysis, a two‐sided Pearson's correlation test was used. ^*^ *P *< 0.05, ^**^ *P *< 0.01, ^***^ *P *< 0.005. and the exact *P* values are indicated in the figures.

## CONFLICTS OF INTEREST

The authors declare no competing interests.

## AUTHOR CONTRIBUTIONS

Meizhang Li designed and performed the experiments and analysed the data. Buddhadeb Dawn directed the Midwest Stem Cell Therapy Center and Rupal Soder generated, characterized, and maintained clinical grade WJMSCs. Neil Dunavin, Sunil Abhyankar, and Siddhartha Ganguly took charge of clinical trial and collected clinical samples. Haitham Abdelhakim, Camille V. Trinidad, and Mitch Braun discussed the hypothesis and contributed to data analysis related to in vitro T cell activation. Harsh B. Pathak and Ziyan Pessetto discussed the hypothesis and contributed to data analysis related to clinical trial. Clayton Deighan performed the ExoView to count exosome number in human plasm samples. Joseph McGuirk, Neil Dunavin, and Andrew K. Godwin coordinated the project, and Meizhang Li, Neil Dunavin, and Andrew K. Godwin interpreted data and wrote the manuscript. All authors reviewed and approved the manuscript. Correspondence and requests for materials should be addressed to Andrew K. Godwin (agodwin@kumc.edu).

## Supporting information

Supplementary information
