# Supplementary material for: WJMSC‐derived small extracellular vesicle enhance T cell suppression through PD‐L1
Source: J Extracell Vesicles. 2021 Feb 8;10(4):e12067. doi: 10.1002/jev2.12067 (PMC7869022; doi:10.1002/jev2.12067)
Supplement: Supplementary file 1 — Supplementary information [file JEV2-10-e12067-s001.docx]

**Supplemental Figures**

**
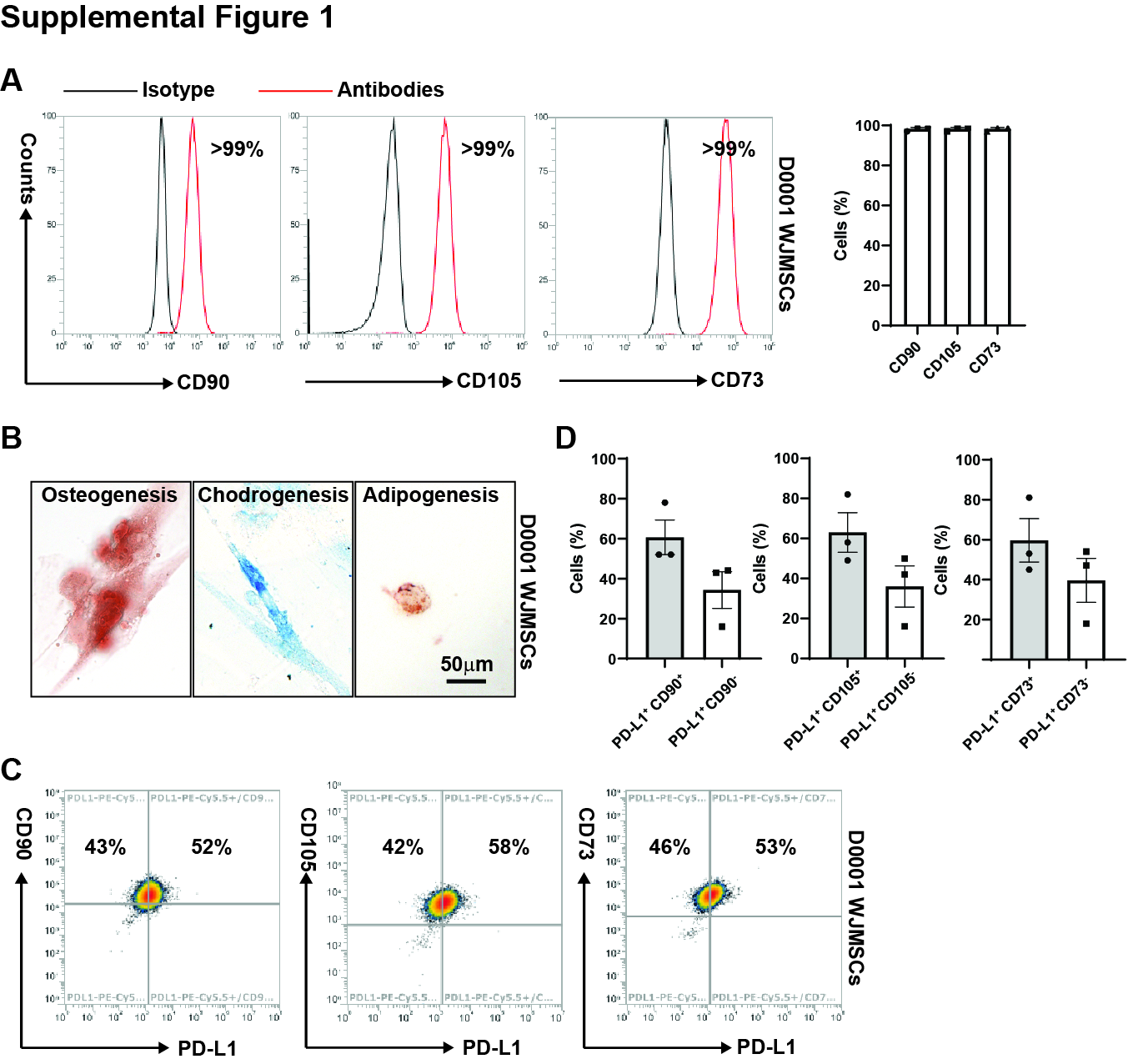
**

**Supplemental Figure 1. Expression of PD-L1 on clinical grade WJMSCs.** (**A**) Representative flow chars (left) of cell surface expression of stem cell markers CD90, CD105 and CD73 on three clinical grade WJMSCs (D0001, D0002 and D0003) and quantitative analysis (right, n=3). (**B**) *In vitro* differentiation of WJMSCs (D0001) into bone cells (left), cartilage cells (middle) and fat cells (right). Scale bar, 50 µm. (**C**-**D**) Co-expression of PD-L1 on CD90^+^, CD105^+^ and CD73^+^ WJMSCs, measured by flow cytometry (**C**) and quantitation (**D**, n=3). Data are mean ± s.e.m. (**A**, **D**).


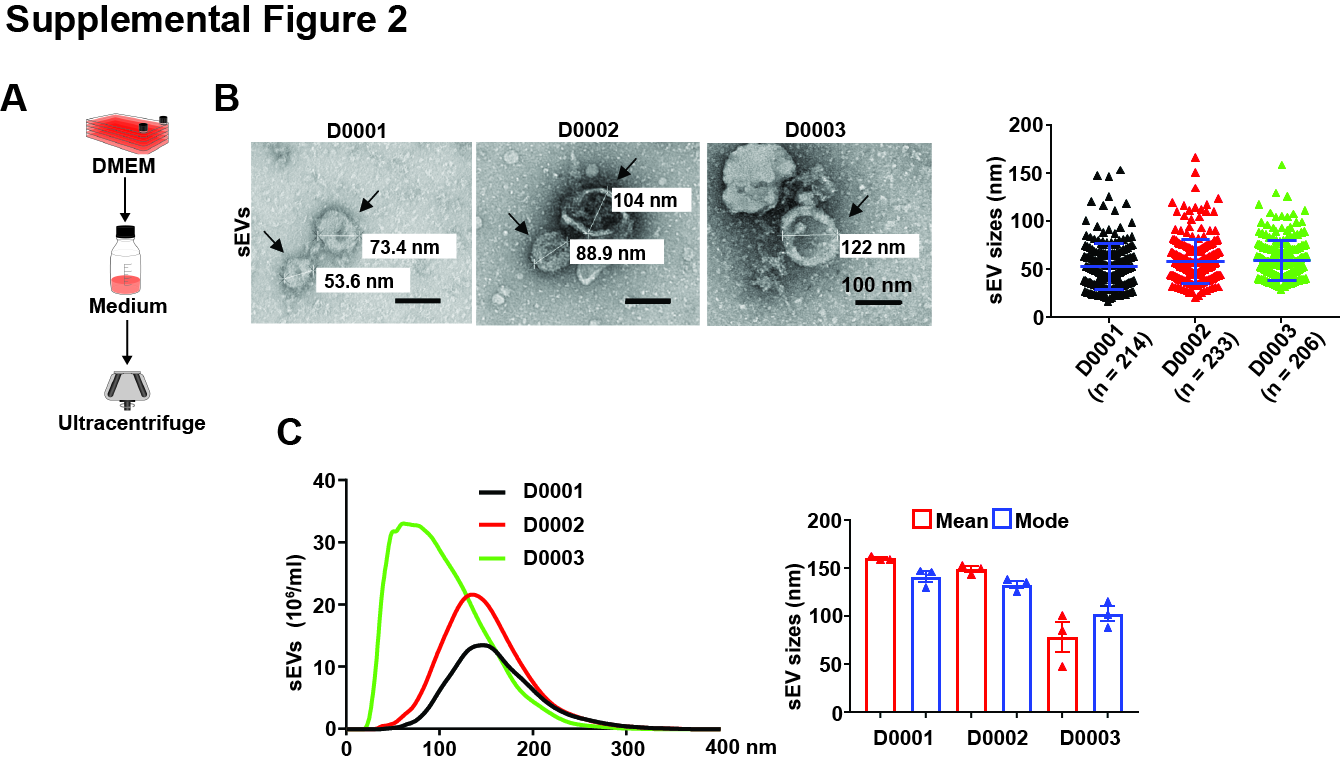


**Supplemental Figure 2. Isolation and characterization of WJMSC-derived sEVs.** (**A**) Schematic of ultracentrifuge-based enrichment of WJMSC sEVs (see Methods). (**B**) Representative TEM imaging of sEVs isolated from three clinical grade WJMSCs (left, arrows) and their mean sizes (right). Scale bar: 100 nm. (**C**) Quantitation of particle numbers (left, n = 3) and the average sizes (right, n=3) for WJMSC sEVs, measured by Nanoparticle Tracking Analysis (NTA). Data are mean ± s.e.m. (**B**-**C**).

**Supplemental Figure 3. Quantifying PD-L1 on the WJMSC-derived sEVs via BLI.** (**A**) Binding curves showing the real-time molecular binding activities between PD-L1-Ab (red curves) or Isotype (blue curves) and sEV-PD-L1, measured by BLI. (**B**) Establishment of standard binding curves using 0.125-1.0 mg/ml soluble recombinant human PD-L1 protein. (**C**) Quantitation of sEV-PD-L1 based on the standard curves from **B**. PD-L1-Ab, detection antibody for human PD-L1 (**A**) and hPD-L, human recombinant PD-L1 protein (**B**). (**D**-**G**) PD-L1 detected on WJMSC-derived exosomes via qEVoriginal/70nm columns (Izon Science, USA). Total protein quantity (**D**); Particle numbers (**E**) and mode size (**F**) measured by Nanoparticle Tracking Analysis (NTA); PD-L1 was detected by BLI on the surface of WJMSC-derived exosomes (**G**). Data are mean ± s.e.m. (n = 3) and analyzed by unpaired one-tailed Student’s t-test (**C**).

**Supplemental Figure 4. In vitro TCR-mediated CD4^+^ TCA and expression of PD1 protein on the activated CD4^+^ T cells.** (**A**) Schematic of *in vitro* TCR-mediated activation of CD4^+^ T cells demonstrated by the increased cell surface CD154. Ab, antibodies; TCRs, T cell receptors; PD1, Programmed cell death protein 1. (**B**) Representative flow cytometry gates of CD154 on the unstimulated CD4^+^ T cells. SSC, side scatter; FSC, forward scatter. (**C**-**D**) Flow cytometry (n = 3) showing the increased expressions of CD154 (**C**) and PD1 (**D**) on the stimulated CD4^+^ T cells. Iso, isotype; CD154-Ab, anti-human CD154 antibody; PD1-Ab, anti-human PD1 antibody; (**E**) Pearson correlation between CD154 and PD1 on unstimulated (blue circle) and stimulated (red circle) CD4^+^ T cells (n = 47). (**F**) Co-expression of CD154 and PD1 on the stimulated CD4^+^ T cells, measured by flow cytometry (left) and quantitative analysis (right, n = 6). (**G**) Immunofluorescence showing the expression of PD1 in either stimulated (arrow) or unstimulated (arrowhead) CD3^+^ T cell. Cells were stained by antibodies anti-human CD3 (green) and PD1 (red), and counterstained by DAPI (blue). Scale bar: 25 µm. PBMCs were stimulated with (Unstim) or without (Stim) CD3/CD28 Dynabeads at a dilution of 1:1 ratio (**A**-**G**). Data are mean ± s.e.m. and analyzed by one-way ANOVA (**C**-**F**).

**Supplemental Figure 5. WJMSC sEVs inhibit TCR-mediated CD8^+^ TCA.** (**A**) Schematic of TCR-mediated CD8^+^ T cell activation (IFN-γ^+^) stimulated by 1 ng/ml CMV-pp65 peptide and incubated with 3.125-15 μg/ml WJMSC sEVs. CMV-pp65, PepTivator cytomegalovirus (cmv) pp65 peptide; MHC II, major histocompatibility complex class II; TCR, t cell receptor; IFN-γ, interferon γ. (**B**) Representative flow cytometry gates of IFN-γ in the unstimulated CD8^+^ T cells. SSC, side scatter; FSC, forward scatter. (**C**-**D**) Flow cytometry showing the inhibitory effects of WJMSC sEVs on TCR-mediated CD8^+^ TCA (**C**) and quantitative analysis (**D**, n =3). Data are mean ± s.e.m and analyzed by one-way ANOVA. Experiments were independently repeated three times (**C**).


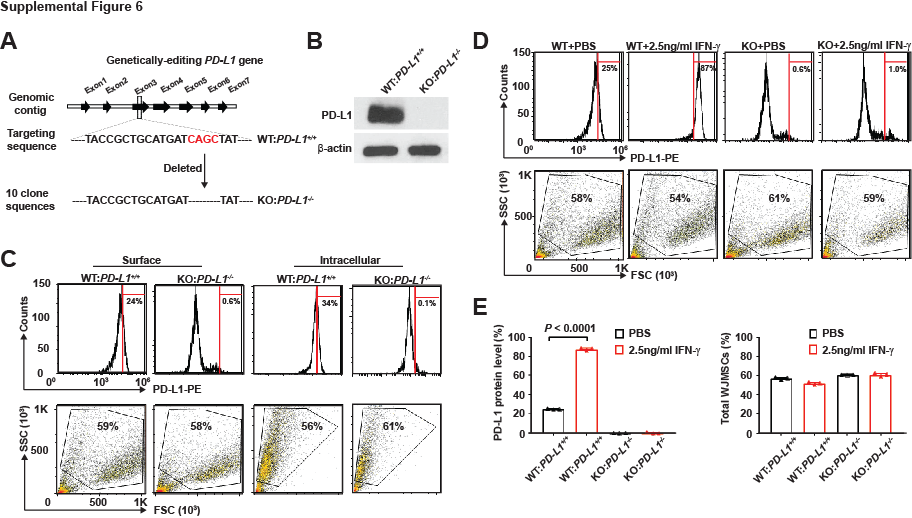


**Supplemental Figure 6. Genetic disruption of *PD-L1* gene in WJMSCs.** (**A**) Schematic of CRISPR-Cas9 gene editing site (-TACCGCTGCATGATCAGCTAT-) within the 3^rd^ exon of *PD-L1* gene in D0001 WJMSCs (see Methods). Editing-site-bearing genomic DNA was cloned into TA cloning vectors and a homozygous “-CAGC-” deletion in the editing site was identified by sequencing the cloned DNA fragment. WT: *PD-L1^+/+^*, PD-L1 wild type; KO: *PD-L1^-/-^*; PD-L1 knockout. (**B**-**C**) Loss of PD-L1 protein in KO WJMSCs verified by immunoblotting (**B**) and flow cytometry (**C**). All lanes were loaded with the same amount of total protein (**B**). (**D**-**E**) Flow cytometry showing that 2.5 ng/ml IFN-γ did not induce PD-L1 on *PD-L1^-/-^* WJMSCs (**D**) and quantitative analysis (**E**, n=3). SSC, side scatter; FSC, forward scatter. Data are mean ± s.e.m. and analyzed by one-way ANOVA (**E**).

**Supplemental Figure 7. IFN-γ failed to induce PD-L1 in *PD-L1^-/-^* WJMSCs.** Immunofluorescence showing PD-L1 protein in WT and *PD-L1^-/-^* WJMSCs induced by 2.5 ng/ml IFN-γ. WJMSCs were induced by PBS (A, A’, A’’, C, C’, C’’) or IFN-g (B, B’, B’’, D, D’, D’’). WT WJMSCs (A, A’, A’’, B, B’, B’’) and *PD-L1^-/-^* WJMSCs (C, C’, C’’, D, D’, D’’). IgG control staining for WT WJMSCs induced by PBS (E, E’, E’’). Top row, merged images; Middle row, DAPI counterstaining (arrows); Bottom row, PD-L1 staining (arrowheads). Scale bar: 100 µm.

**Supplemental Figure 8. Normal phenotype of sEVs isolated from *PD-L1^-/-^* WJMSCs.** (**A**) Immunoblotting showing the loss of PD-L1 protein in the sEVs isolated from the *PD-L1^-/-^* WJMSCs. (**B**-**C**) Loss of PD-L1 protein on the surface of *PD-L1^-/-^* WJMSC sEVs, measured by immunogold TEM (**B**, scale bar: 100 nm) and BLI (**C**). (**D**-**E**) Normal yield and sizes of PD-L1-deficient sEVs compared to the wild types’, verified by Nanoparticle Tracking Assay (NTA, **d**) and Transmission Electron Microscopy (TEM, scale bar: 100nm, **E**). (**F**-**G**) 2.5 ng/ml IFN-γ did not induce the sEV-PD-L1 release from *PD-L1^-/-^* WJMSCs, confirmed by immunoblotting (**F**) and ELISA (**G**). All lanes were loaded with the same amount of total protein (**A**, **F**). Data are mean ± s.e.m. (n = 3) and analyzed by one-way ANOVA (**C**-**E**, **G**).

**Supplemental Figure 9. Particle numbers of plasma exosomes in aGvHD patients with WJMSC infusion.** (**A**) Schematic of exosomes captured by ExoView chips from aGvHD patients’ plasma samples and stained with antibodies anti-exosome biomarkers. Ab, antibodies for both capturing and detection. (**B**) Representative images showing the expression of exosomal biomarkers CD81^+^ (green channel), CD9^+^ (blue channel) and CD63^+^ (red channel) on the captured exosomes. (**C**-**F**) Quantitative analysis of plasma exosomes captured by antibodies anti-human CD81 (**C**), CD63 (**D**), CD9 (**E**), and IgG (**F**). Total, CD81^+^, CD9^+^, and CD63^+^ exosomes are individually indicated by the grey, green, blue, and red bars (**C**-**F**). Data are mean ± s.e.m. (n=3) and analyzed by unpaired one-tailed Student’s t-test (**C**-**F**). **P* <0.05, ***P* <0.01, ****P* <0.005.
